# Circulating adipokine levels and preeclampsia: A bidirectional Mendelian randomization study

**DOI:** 10.3389/fgene.2022.935757

**Published:** 2022-08-22

**Authors:** Xiaoyan Chen, Zhaoming Liu, Jingen Cui, Xiaolan Chen, Jing Xiong, Wei Zhou

**Affiliations:** Department of Obstetrics, Chongqing Health Center for Women and Children, Women and Children’s Hospital of Chongqing Medical University, Chongqing, China

**Keywords:** adipokine, preeclampsia, Mendelian randomization, genetic epidemiology, single nucleotide polymorphism

## Abstract

**Background:** Several observational studies have demonstrated that significantly rising circulating adipokine levels are pervasive in preeclampsia or eclampsia disorder (or preeclampsia toxemia (PET)). However, it remains unclear whether this relationship is causal. In this study, we sought to elucidate the causal effects of circulating adipokine levels on PET.

**Methods:** Summary-level data and independent genetic variants strongly associated with common adipokine molecule (adiponectin, leptin, resistin, sOB-R, and PAI-1) levels were drawn from public genome-wide association study (GWASs). Additionally, the corresponding effects between instrumental variables and PET outcomes were acquired from the FinnGen consortium, including 4,743 cases and 136,325 controls of European ancestry. Subsequently, an inverse-variance weighted (IVW) approach was applied for the principal two-sample Mendelian randomization (MR) and multivariable MR (MVMR) analyses. Various complementary sensitivity analyses were then carried out to determine the robustness of our models.

**Results:** The results of the IVW method did not reveal any causal relationship shared across genetically predisposed adipokine levels and PET risk (for adiponectin, OR = 0.86, 95% CI: 0.65–1.13, *p* = 0.274). Additionally, no significant associations were identified after taking into account five circulating adipokines in MVMR research. Complementary sensitivity analysis also supported no significant associations between them. In the reverse MR analysis, genetically predicted PET risk showed a suggestive association with elevating PAI-1 levels by the IVW method (Beta = 0.120, 95% CI: 0.014, 0.227, *p* = 0.026). Furthermore, there were no strong correlations between genetic liability to PET and other adipokine levels (*p* > 0.05).

**Conclusion:** Our MR study did not provide robust evidence supporting the causal role of common circulating adipokine levels in PET, whereas genetically predicted PET may instrumentally affect PAI-1 levels. These findings suggest that PAI-1 may be a useful biomarker for monitoring the diagnosis or therapy of PET rather than a therapeutic target for PET.

## Introduction

Preeclampsia or eclampsia, also known as preeclampsia toxemia (PET), is the leading cause of maternal as well as fetal morbidity and mortality among the hypertensive disorders of pregnancy (Rana et al., 2019). Although their incidence varies widely, it is estimated to complicate approximately 4.5% of all pregnancies in the Western world (Abalos et al., 2013). The etiology of PET is probably heterogeneous and not completely clarified, but it is believed that a variety of inflammatory and angiogenic mediators affecting vascular endothelial function and reactivity are attributed to the phenotype of preeclampsia (Bellos et al., 2018). Continued efforts have been made to combat PET; however, there is still a long way to reduce the disease burden of PET. One of the key points is early screening (or diagnosis) based on circulating biomarkers.

It has been well established that adipose-derived adipokines have been assumed to be involved in preeclampsia pathogenesis (Gutaj et al., 2020). As indispensable members of adipokines (a type of immune molecules and inflammatory mediators), common adipokines leptin and resistin have been increasingly studied in the context of risk of PET onset and progression (Masuyama et al., 2010; Daskalakis et al., 2020; Banjac et al., 2021). For example, previous epidemiological studies reported increased serum leptin levels in PET patients and elevated levels of this protein in preeclamptic placentas (Kalinderis et al., 2015; Taylor et al., 2015). Meanwhile, several observational studies indicate that circulating levels of leptin are lower or similar in women with clinical PET than in their normotensive counterparts (Martinez-Abundis et al., 2000; Laml et al., 2001; Doster et al., 2016; Kharb et al., 2017). A recent meta-analysis suggested that patients with PET have a significantly higher leptin level than a group of healthy controls; however, typically only limited research with a small number of patients are available (Shahid et al., 2022).

Conclusive causal relationships remain unestablished on the basis of observational evidence alone due to reverse causation and residual confounding factors, such as incomplete adjustment for confounders, the absence of high-quality evidence, and relatively small sample sizes of trials. Mendelian randomization (MR) is an epidemiological method that uses genetic variants (single nucleotide polymorphisms, SNPs) as instrumental variables (IVs) and could mimic biological effects of clinical biomarkers, and it is less susceptible to the aforementioned shortcomings (Lawlor et al., 2008; Davey Smith and Hemani, 2014). As germline variants in parental genes tend to be randomly distributed to offspring in meiosis during gametogenesis and at conception, MR analysis can eliminate residual confounding from environmental factors and strengthen the causal inference (Burgess et al., 2019). Based on the existing genome-wide association study (GWAS) databank, MR has been widely used to quantitatively assess the effect of circulating adipokine levels on the risk of various diseases, such as cancers, cardiovascular diseases, and auto-inflammatory disorders (Dimou et al., 2021; Harroud et al., 2021; Chen et al., 2022).

However, the potential causal relationship between circulating adipokines and PET has scarcely been explored using the MR approach. Hence, we conducted a bidirectional MR study to test the association of five genetically predicted adipokine biomarkers [adiponectin, leptin, resistin, soluble leptin receptor (sOB-R), and plasminogen activator inhibitor-1 (PAI-1)] with the risk of PET. In addition, we also conducted multivariable MR (MVMR) analysis to determine whether circulating adipokine levels are associated with PET outcomes independently of each other.

## Methods

### Study design

**FIGURE 1:**
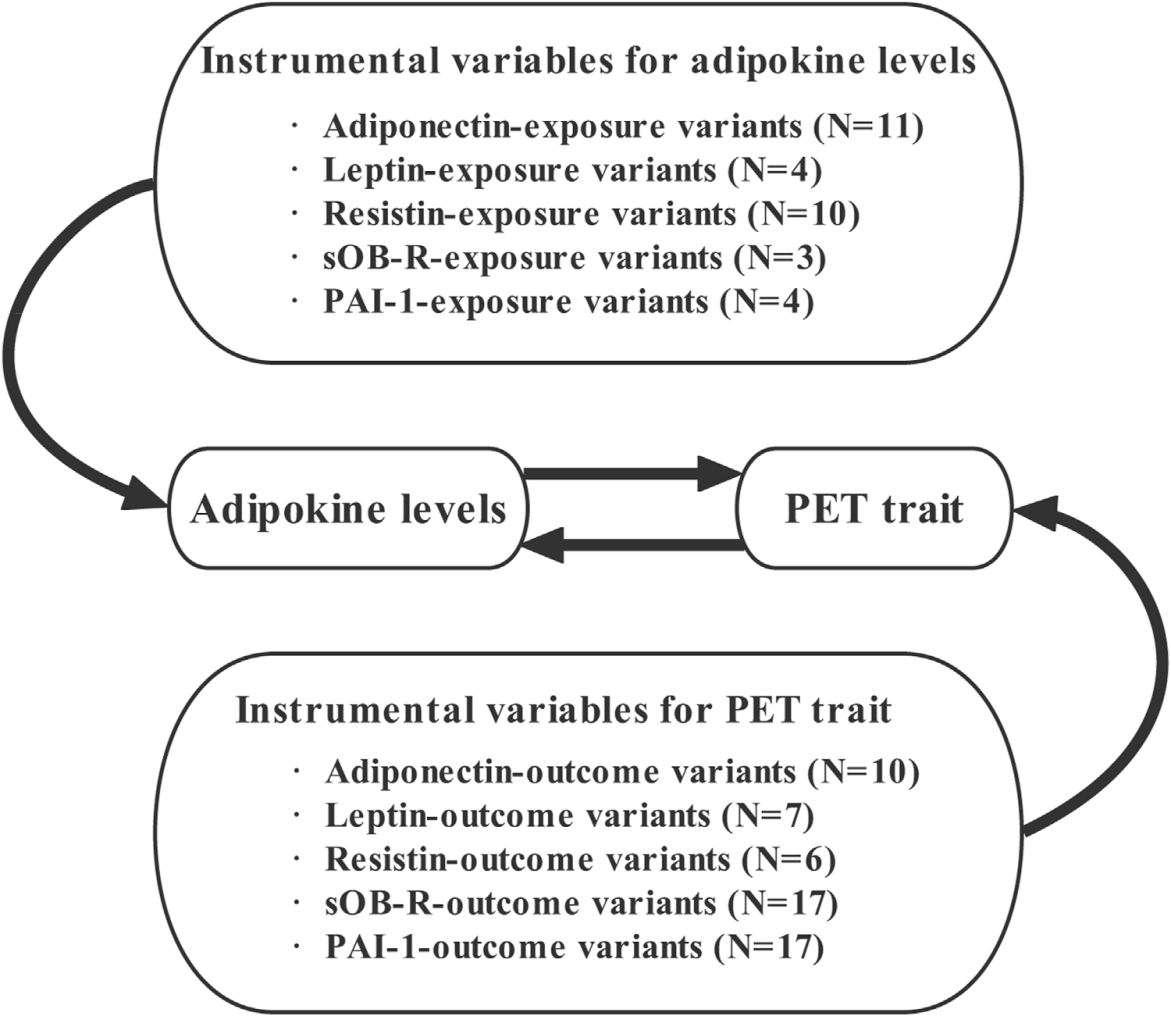
Overview of our study design aimed at revealing the bidirectional relationships between circulating adipokines and PET. Abbreviations: N, number of SNPs; PET, preeclampsia or eclampsia.

To allow for an adequate number of SNPs to be included in our MR analysis, we thoroughly relaxed the GWAS *p*-value threshold of *p* < 5 × 10^–6^ for the five adipokine biomarkers. We performed a two-sample MR study using the publicly available summary-level GWAS datasets on adipokines and PET. The validity of MR analysis relies on three critical assumptions: 1) the selected IVs should be closely related to the exposure factor. The IV–exposure strength of genetic instruments was assessed from the F statistic using an approximation (Bowden et al., 2016). If F > 10, there is sufficient strength to avoid a problem of weak instrument bias in the two-sample model (Burgess and Thompson, 2011); 2) each IV must influence the outcome only through exposure factors rather than another pathway; 3) the selected IVs need to be independent of unobserved confounders of the exposure–outcome relationship (Lawlor et al., 2008). Additionally, ethical approval or consent to participate was documented in the original publications. The overview of the study design is presented in Figure 1.

### Data sources

To be more specific, selection of strong adipokine-level estimates of correlations between genetic variants can be obtained from several previously published GWAS datasets of European ancestry (1,338 ≤ N ≤ 39,883) (Dastani et al., 2012; Huang et al., 2012; Kilpelainen et al., 2016; Suhre et al., 2017; Folkersen et al., 2020). A generalized linear mixed model was applied to test for association with genotyped or imputed SNPs that were adjusted for age, sex, and body mass index (BMI). Within each study, circulating adipokine levels were natural logarithm transformed to approximate normal distribution. Further details concerning the relevant genetic information could be acquired from the aforementioned research studies.

Summary data for PET genetic association estimates were extracted from the FinnGen consortium (R6) (https://finngen.gitbook.io/documentation) with 4,743 cases and 136,325 controls of European individuals. Details of population characteristics are available in the original publications and websites. There is no sample overlap between the exposure and outcome datasets. All datasets for the MR analysis are summarized in Table 1. To avoid a possible bias in the estimates of linkage disequilibrium (LD), we attempted to exclude SNPs that met the condition *r* ^2^ < 0.001. When multiple tagging IVs were in strong LD with each other, only the corresponding IV with the smallest *p* value was appropriately chosen. Additionally, any specifically requested SNPs that were not available in outcome datasets would be replaced with proxies in LD of *r* ^2^ > 0.8 or excluded from the MR if no eligible proxies were identified. We also manually searched secondary phenotypes of each selected SNP and its proxy in PhenoScanner V2 (http://www.Phenoscanner.cam.ac.uk/) to rule out the possible influence of pleiotropic effects. The effects of ambiguous SNPs being palindromic with intermediate allele frequencies (where “palindromic SNPs” refer to SNPs with A/T or G/C alleles and “intermediate allele frequencies” refer to 0.01 < allele frequency < 0.30) would be discarded in the subsequent two-sample MR analysis.

**TABLE 1:** Basic characteristics for selected summary-level GWASs applied in MR study.

Trait	First author/consortium	Sample size	Cases	Ethnicity	Sex	References
Adiponectin	Dastani Z	39,883	NA	European	Males and females	[23]
Leptin	Kilpelainen TO	32,161	NA	European	Males and females	[24]
Resistin	Folkersen L	30,931	NA	European	Males and females	[25]
sOB-R	Suhre K	1,338	NA	European	Males and females	[26]
PAI-1	Huang J	30,395	NA	European	Males and females	[27]
PET	FinnGen team	141,068	4,743	European	Females	^a^

a https://finngen.gitbook.io/documentation

Abbreviation: NA, not available.

### Bidirectional Mendelian randomization

To examine the possibility that reverse causality exists in these studies, a bidirectional two-sample MR analysis between genetically predicted PET and circulating adipokines was performed using the same approach as described earlier. Since summary-level data for outcome (sOB-R and PAI-1) were not obtained from the corresponding GWASs, other GWAS summary data for these two adipokines were chosen from the MRC IEU open GWAS platform (https://gwas.mrcieu.ac.uk/); the GWAS IDs corresponding to sOB-R and PAI-1 were “prot-a-1724” and “prot-a-2696,” respectively.

### Statistical analysis

The fixed-effects inverse-variance weighted (IVW) meta-analysis based on the Wald ratio method was applied to explore two-sample MR estimates of the associations between the levels of the five adipokines and PET(28). The IVW method, including all valid IVs, would provide the most accurate assessments. We further applied MVMR (Burgess and Thompson, 2015) to dissect the influence of potential adipokine levels on causal estimates. Additionally, a series of complementary sensitivity analyses, such as maximum likelihood, MR-Egger regression, weighted median, and MR-robust adjusted profile score (MR-RAPS) methods, were introduced to produce a consistent causal estimate (Pierce and Burgess, 2013; Bowden et al., 2015; Hartwig et al., 2016; Zhao et al., 2019). Moreover, estimating an intercept with *p* < 0.05 in MR-Egger regression is akin to allowing for the possibility of additional horizontal pleiotropy. MR-pleiotropy residual sum and outlier (MR-PRESSO) test was applied to detect potential outlying SNPs and provide causal estimates after the removal of outliers (Verbanck et al., 2018). We also applied radial IVW for a better visualization of the regression estimates. The statistical heterogeneity among the estimates of IVs in the IVW method was assessed using Cochran’s Q test, and *p* values less than 0.05 were considered statistically significant. To address multiple testing, a Bonferroni-corrected *p* value of 0.005 (0.05/10 adipokines) was considered significant, with a *p* value < 0.005 or < 0.05 was regarded as suggestive. Furthermore, a “leave-one-out” sensitivity analysis was performed to determine the potential effect of SNPs on the causal estimates. Power calculations were performed based on a web tool available at https://sb452.shinyapps.io/power.

All these analyses were implemented in the R software (version 4.1.0, using the “TwoSampleMR,” “RadialMR,” and “MR-PRESSO” R packages; R Foundation for Statistical Computing, Vienna, Austria).

## Results

A detailed description of the selected SNPs, including effect allele (EA), EA frequency, and effect sizes on adipokines and PET, was thoroughly retrieved from the original literatures. All selected SNPs did not have a potent LD calculation (*r* ^2^ < 0.001). For genetically predicted exposure level, power calculations for our bidirectional two-sample MR analyses were performed. All statistical power calculations for our MR estimates were less than 80%, except for the effect of PAI-1 level on PET (Table 1).

### Circulating adipokine levels and PET

Summary information about the selected SNPs for the five adipokine levels is available in Supplementary Table S2. In this study, 6 SNPs (rs601339, rs7133378, rs13081028, rs10773049, rs825453, and rs8182584) for adiponectin level, five SNPs (rs199752470, rs34861192, rs7746716, rs4134826, and rs3745367) for resistin level, and 1 SNP (rs17412403) for sOB-R level were removed due to high LD with other variants or absence from the LD reference panel. Additionally, we eliminated 1 SNP (rs10487505 related to leptin) for being palindromic with intermediate allele frequencies. Thus, the remaining 11 SNPs regarded as IVs for adiponectin level, four SNPs for leptin level, 10 SNPs for resistin level, three SNPs for sOB-R level, and four SNPs for PAI-1 level were included in further MR analysis. The F statistics of all selected SNPs ranged from 18 to 225, demonstrating that there is no bias due to weak instruments. Moreover, the variance explained in circulating adipokine levels by the genetic instruments was 6.04%, 1.69%, 4.67%, 9.06%, and 1.87% for adiponectin, leptin, resistin, sOB-R, and PAI-1 levels, respectively.

**FIGURE 2:**
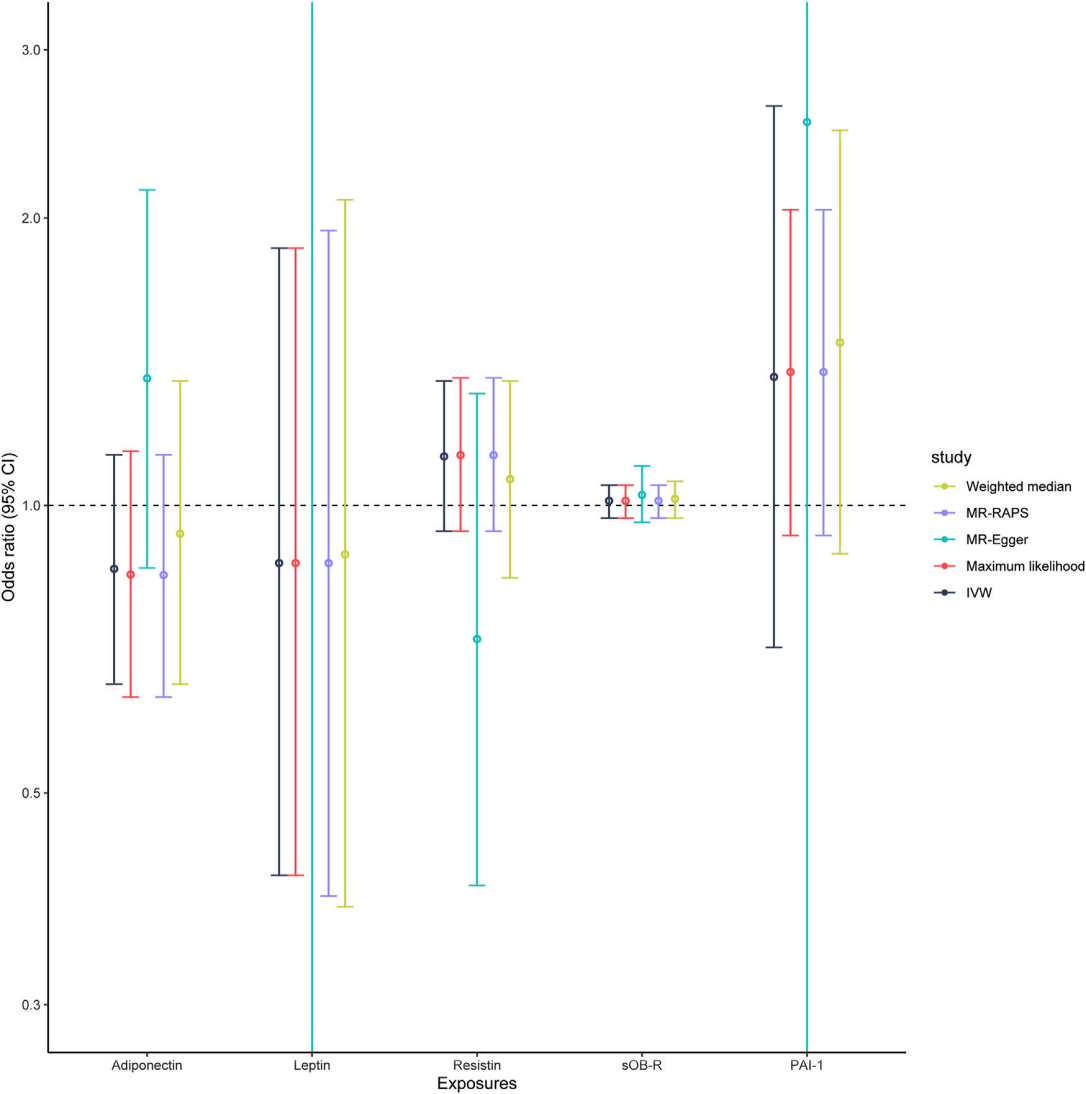
Associations of genetically predicted circulating adipokine levels with PET according to different Mendelian randomization methods. Abbreviations: IVW, inverse-variance weighted; MR-Egger, Mendelian randomization Egger; MR-RAPS, Mendelian randomization robust adjusted profile score; OR, odds ratio; 95% CI, 95% confidence interval.

As shown in Table 2, genetically predicted levels of circulating adipokines were not associated with the risk of PET (adiponectin, OR = 0.86, 95% CI: 0.65–1.13, *p* = 0.274; leptin, OR = 0.87, 95% CI: 0.41–1.86, *p* = 0.72; resistin, OR = 1.13, 95% CI: 0.94–1.35, *p* = 0.199; sOB-R, OR = 1.01, 95% CI: 0.97–1.05, *p* = 0.599; and PAI-1, OR = 1.36, 95% CI: 0.71–2.62, *p* = 0.354, Figure 2) using the IVW method. Moreover, such findings were undeviating from several complementary MR analyses (*p* > 0.05). When five circulating adipokines were included together in the MVMR analysis (Supplementary Table S3), all its estimations with PET hardly surpassed the Bonferroni-corrected threshold (*p* > 0.05). Cochran’s Q statistics showed evidence of slight heterogeneity based on genetically predicted SNPs of PAI-1 level (*p* = 0.032), and the associations for PAI-1 were mainly driven by rs6976035 located near the ACHE region in sensitivity analyses. The results of the leave-one-out method demonstrated that the links between other adipokines and PET risk were not driven by SNPs, which indicated that MR results were reliable (Supplementary Figure S1). Moreover, the MR-Egger intercept test detected no evidence of directional pleiotropy (*p* > 0.05). No outlier SNPs were observed in the MR-PRESSO analysis (data not shown). Additionally, the IVW Radial MR results delineated that no instrumental variables of adipokines that carry large effect sizes on PET could potentially be outliers.

**TABLE 2:** Two-sample MR estimates of associations between genetically predicted circulating adipokine levels and PET.

Exposure	Method	SNPs	OR	95% CI	*p*	Q statistic	P-heterogeneity	P-intercept
Adiponectin	IVW	11	0.86	0.65–1.13	0.274	15.19	0.125	
	Maximum likelihood	11	0.85	0.63–1.14	0.279			
	MR-Egger	11	1.36	0.86–2.14	0.218			0.510
	Weighted median	11	0.93	0.65–1.35	0.718			
	MR-RAPS	11	0.85	0.63–1.13	0.252			
Leptin	IVW	4	0.87	0.41–1.86	0.720	0.097	0.992	
	Maximum likelihood	4	0.87	0.41–1.86	0.720			
	MR-Egger	4	3.62	1.15e-04–1.14e+05	0.830			0.812
	Weighted median	4	0.89	0.38–2.09	0.787			
	MR-RAPS	4	0.87	0.39–1.94	0.735			
Resistin	IVW	10	1.13	0.94–1.35	0.199	15.62	0.075	
	Maximum likelihood	10	1.13	0.94–1.36	0.194			
	MR-Egger	10	0.72	0.40–1.31	0.316			0.154
	Weighted median	10	1.07	0.84–1.35	0.600			
	MR-RAPS	10	1.13	0.94–1.36	0.194			
sOB-R	IVW	3	1.01	0.97–1.05	0.599	1.193	0.551	
	Maximum likelihood	3	1.01	0.97–1.05	0.599			
	MR-Egger	3	1.03	0.96–1.10	0.605			0.706
	Weighted median	3	1.02	0.97–1.06	0.497			
	MR-RAPS	3	1.01	0.97–1.05	0.601			
PAI-1	IVW	4	1.36	0.71–2.62	0.354	8.78	0.032	
	Maximum likelihood	4	1.38	0.93–2.04	0.108			
	MR-Egger	4	2.52	0.05–118.8	0.684			0.779
	Weighted median	4	1.48	0.89–2.47	0.131			
	MR-RAPS	4	1.38	0.93–2.04	0.105			

Abbreviations: IVW, inverse-variance weighted; MR-Egger, Mendelian randomization Egger; MR-RAPS, Mendelian randomization robust adjusted profile score; SNP, single nucleotide polymorphism; OR, odds ratio; 95% CI, 95% confidence interval.

A few genetic variants (rs998584 for adiponectin, rs780093 for leptin, rs3087852 for resistin, rs4655537 for sOB-R, and rs11128603 for PAI-1) were associated with other phenotypes at the threshold of genome-wide significance (*p* < 5 × 10^–8^), including different white blood cells, BMI, lipid levels, coronary artery disease, and age at menopause (Supplementary Table S4). Remarkably, all these traits were unlikely to exert any pleiotropic effect on the observed associations between genetically predicted adipokine levels and PET risk.

### PET and circulating adipokine levels

**FIGURE 3:**
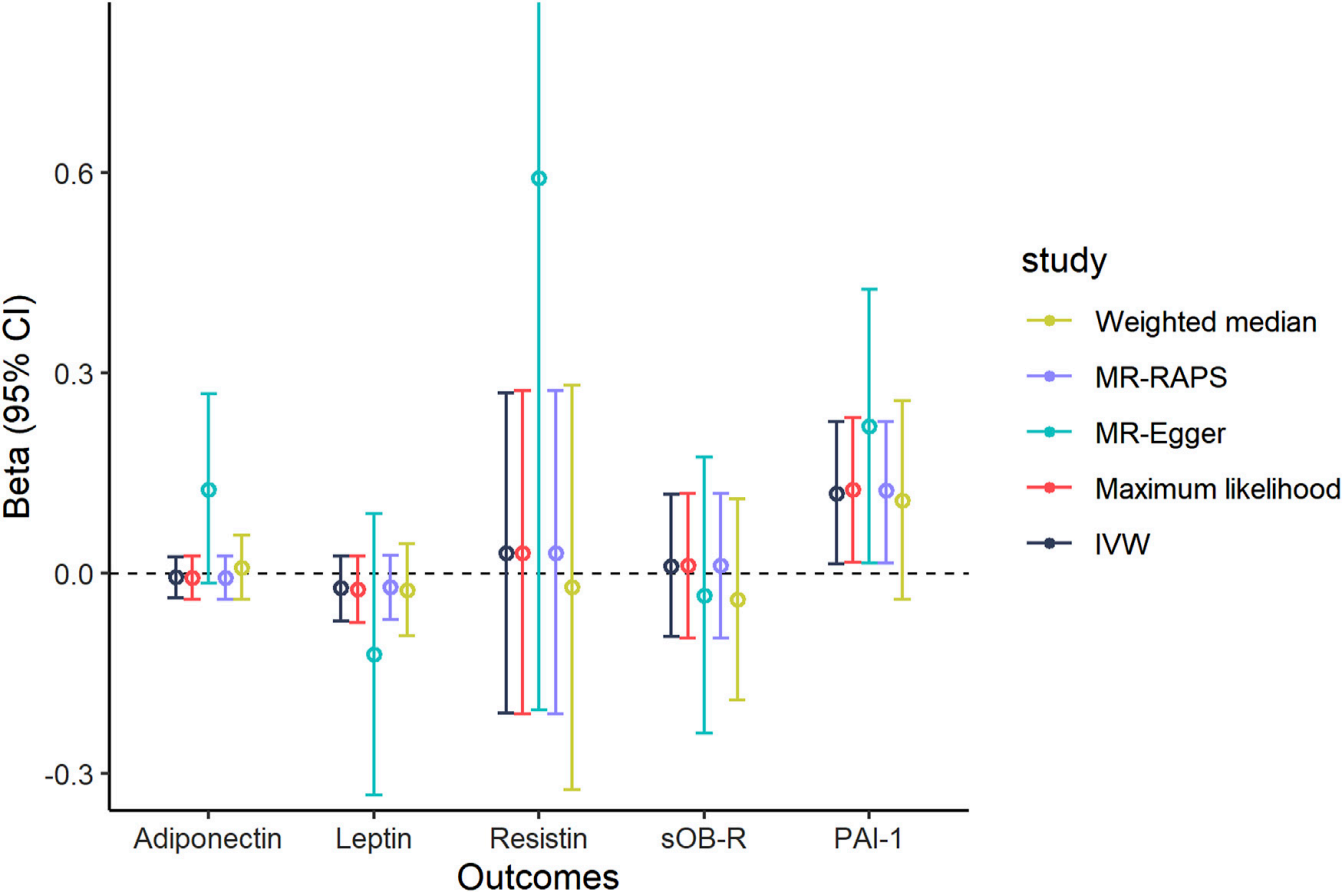
Causal association of genetically predicted PET and circulating adipokine levels according to different Mendelian randomization methods. Abbreviations: IVW, inverse-variance weighted; MR-Egger, Mendelian randomization Egger; MR-RAPS, Mendelian randomization robust adjusted profile score; 95% CI, 95% confidence interval.

To further examine the influence of PET on adipokine concentrations, we conducted another reverse MR design regarding genetical PET trait as exposure and circulating adipokines as outcome. A total of 11 SNPs that were strongly related to PET risk (*p* < 5 × 10^–6^) were identified in our MR findings (Supplementary Table S5). The MR results indicated that there was a suggestive positive link between genetic liability to PET and circulating PAI-1 levels using the IVW, maximum likelihood, MR-Egger, and MR-RAPS methods (IVW, Beta = 0.120, 95% CI: 0.014, 0.227, *p* = 0.026, Figure 3 and Table 3). In addition, null estimates were obtained for the causal effects of PET on other circulating adipokine levels. The leave-one-out analysis also did not demonstrate any evidence that an SNP could drive the overall effect of genetical PET variants on adiponectin levels (Supplementary Figure S2). Similarly, neither indication of a significant intercept for horizontal pleiotropy (intercept = –0.02, *p* = 0.281) nor strong heterogeneity (Cochran’s Q = 13.32, *p* = 0.629) was detected in these MR analyses (Table 3).

**TABLE 3:** Two-sample MR estimates of associations between genetically predicted PET and circulating adipokine levels.

Outcome	Method	SNPs	Beta	95% CI	*p*	Q statistic	P-heterogeneity	P-intercept
Adiponectin	IVW	10	−0.006	(−0.037, 0.025)	0.689	13.78	0.088	
	Maximum likelihood	10	−0.007	(−0.039, 0.026)	0.698			
	MR-Egger	10	0.126	(−0.015, 0.269)	0.117			0.091
	Weighted median	10	0.009	(−0.039, 0.057)	0.720			
	MR-RAPS	10	−0.007	(−0.039, 0.026)	0.656			
Leptin	IVW	7	−0.022	(−0.071, 0.026)	0.363	10.59	0.102	
	Maximum likelihood	7	−0.024	(−0.074, 0.026)	0.346			
	MR-Egger	7	−0.121	(−0.332, 0.090)	0.312			0.381
	Weighted median	7	−0.025	(−0.093, 0.044)	0.477			
	MR-RAPS	7	−0.021	(−0.069, 0.027)	0.346			
Resistin	IVW	6	0.030	(−0.209, 0.270)	0.803	3.34	0.648	
	Maximum likelihood	6	0.031	(−0.211, 0.274)	0.800			
	MR-Egger	6	0.592	(−0.205, 1.391)	0.219			0.221
	Weighted median	6	−0.021	(−0.324, 0.282)	0.891			
	MR-RAPS	6	0.031	(−0.211, 0.274)	0.809			
sOB-R	IVW	17	0.011	(−0.095, 0.118)	0.835	15.42	0.494	
	Maximum likelihood	17	0.012	(−0.097, 0.120)	0.835			
	MR-Egger	17	−0.033	(−0.240, 0.174)	0.757			0.629
	Weighted median	17	−0.039	(−0.190, 0.112)	0.620			
	MR-RAPS	17	0.012	(−0.097, 0.120)	0.837			
PAI-1	IVW	17	0.120	(0.014, 0.227)	0.026	13.32	0.649	
	Maximum likelihood	17	0.125	(0.016, 0.233)	0.024			
	MR-Egger	17	0.221	(0.015, 0.426)	0.042			0.281
	Weighted median	17	0.109	(−0.039, 0.259)	0.164			
	MR-RAPS	17	0.124	(0.015, 0.228)	0.029			

Abbreviations: IVW, inverse-variance weighted; MR-Egger, Mendelian randomization Egger; MR-RAPS, Mendelian randomization robust adjusted profile score; SNP, single nucleotide polymorphism; 95% CI, 95% confidence interval.

## Discussion

To the best of our knowledge, this is the first MR analysis evaluating whether five adipokine levels are related to the risk of PET based on the genetic data from large-scale GWAS datasets. The current MR estimates did not support a causal effect of genetically determined circulating adipokines on PET risk. Moreover, reverse MR analyses indicated that genetic predisposition to PET was possibly associated with the level of circulating PAI-1.

Our findings on the role of circulating adipokine levels in the development of PET seem quite inconsistent across observational studies and interventional trials. Compared to healthy controls, several case–control researches have demonstrated elevated levels of PAI-1 in patients with early-onset PET (Chappell et al., 2002; Wikstrom et al., 2009; Bodova et al., 2011; Rampersaud et al., 2020). A positive association has been confirmed between maternal PAI-1 mRNA expression and the severity of PET in the third trimester, and a linear regression model has evaluated that severe PET is remarkably associated with PAI-1 level (Purwosunu et al., 2007; Elzein et al., 2016). In addition, some researchers argued that there was no difference in PAI-1 expressions between PET individuals and healthy pregnant women (Gow et al., 1984; Ho and Yang, 1992). A previous meta-analysis also did not delineate any significant relationship between the occurrence of PE and PAI-1 4G/5G polymorphism (Morgan et al., 2013). Although accumulating evidences show that increased PAI-1 levels play a pivotal role in PET phenotype, information about the function of PAI-1 during pregnancy complicated with PET are still controversial. Our bidirectional MR analysis has shown the shared genetic architecture between PAI-1 levels and PET and found suggestive evidence of causal relationships between them.

Even though we found no indication of potential causality between other genetically predicted adipokine levels and PET risk, such findings are contradictory with previous observational studies. For adiponectin, multiple researchers confirmed that a significant increase was mapped in adiponectin concentration among PET patients compared to controls (Naruse et al., 2005; Takemura et al., 2007). In contrast, several comparative studies have demonstrated significantly lower levels of adiponectin in PET patients (Ouyang et al., 2007; Herse et al., 2009). Some researchers proved that the involvement of adiponectin in PE pathology was attributed to its function in endothelial nitric oxide synthase activation and nitric oxide synthesis, and both could result in the reduction of blood pressure (Sandrim et al., 2008). With regard to resistin, alterations in its concentration level in PET patients are still poorly understood. Some of the studies concerning PET have concluded that maternal resistin concentrations might be elevated (Seol et al., 2010), some observed reduced resistin level (Christiansen et al., 2015), and others also revealed no significant correlation between PET and non-complicated pregnancies (Hendler et al., 2005). The discrepancy between our MR findings and previous observational studies may partly be due to measurement error, small sample size, and residual confounding. Indeed, further research is required to elucidate the internal association of adiponectin, as well as other adipokines with robust null results in our study, with PET and to identify whether there is potential bias or confounding accounted for previous studies.

Several potential mechanisms of increased PAI-1 synthesis in the pathogenesis of PET and their association have been comprehensively discussed. It has been reported that the hypoinvasion and failed conversion of maternal endometrial spiral arteries in the placenta of patients with PET are related to the increased level of PAI-1 (Redman and Sargent, 2005). Additionally, PET has been commonly recognized as an inflammation-mediated disorder, and inflammatory cytokines, including interleukin 1β (IL-1β), IL-6, vascular endothelial growth factor, and epidermal growth factor, could upregulate the level of circulating PAI-1 (Zhai et al., 2022). Moreover, it has been found that the NF-κB pathway activation in the damaged migration of trophoblasts in PET could ultimately promote the mRNA expression of PAI-1 (Ye et al., 2017). Although the fact whether an increased level of PAI-1 is the leading cause of PET or the meaningful consequence of endothelial and placental dysfunction remains undetermined, our reverse MR results partly supported that upregulations of circulating adipokines involved in PAI-1 level appear to be downstream effects of PET.

As far as we know, there are no related MR analyses that have explored the bidirectional association of circulating adipokine levels with the risk of PET. A recent MR research revealed the causal role of resistin in the development of cardiovascular disease, which probably acted through blood pressure (Chen et al., 2022). Another MR study provided evidence for a potential causal link between leptin level and blood pressure among smokers (Shen et al., 2020). In our MR study, we incorporated the largest and most comprehensive datasets to investigate a causal association between genetically determined circulating adipokine concentrations and risks of PET. In addition, we performed various sensitivity analyses and investigated potential associations with secondary phenotypes of interest. Our results were similar and robust, suggesting that pleiotropy did not markedly influence our findings. However, our present findings cannot rule out the possibility of a protective or detrimental role for adipokines in PET etiology, which suggests that the function of adipokines in the causal pathway of this disease is likely to be small.

The present MR design was introduced primarily to avoid the bias of observational studies on the association of genetically predicted adipokine levels with PET risk in large-scale individuals. Several important advantages deserve special emphasis. First, a predominant strength of the bidirectional MR method is benefited to reduce bias from unmeasured residual confounding, reverse causality, and measurement error. Second, the most comprehensive GWAS-identified SNPs were regarded as the IV model. In addition, all selected IVs (F > 10) were robustly associated with the exposure factor, suggesting that there were no evidence of weak estimate. Third, several sensitivity analyses and potential pleiotropic validation imply the decreased probability of bias. Thus, our findings provide strong evidence in support of the genetic assessment between adipokine levels and PET susceptibility.

Notwithstanding the explicit strengths, some limitations in our study should be noted. First, the statistical power might be low for several analyses due to limited case numbers. Thus, the inadequate power might be the reason for the null results. Second, the information for our research was obtained from publicly available GWASs’ summary-level datasets. However, detailed demographic characteristics and clinical manifestations (such as smoking status and PET progress) of subjects were not available to perform risk-stratified and conventional subgroup analysis. Third, our population confinement to European descent could help minimize the impact of ethnic structure bias; however, our findings might not generalize to other populations. Fourth, it is difficult for us to make a distinction between preeclampsia and eclampsia in our MR analyses. Fifth, we cannot completely rule out the relationship of sOB-R level with PET risk, since this might be due to the relatively small sample size. Sixth, our research did not acquire enough SNPs regarding plasma adipokine levels as IVs, resulting in only a limited proportion of variation to be explained and no adequate power to reach a significant threshold. Additionally, we acknowledged that MR methods used in our research cannot address commonly seen pleiotropy and sample structure problems in such causal inference studies. Genome-wide MR methods such as Causal Analysis Using Summary Effect Estimates (Morrison et al., 2020) and constrained maximum likelihood and model averaging (Xue et al., 2021) using weak IVs may relieve the concern for possible false-positive causal findings. Seventh, the effects of PET exposure on five plasma adipokine biomarkers could vary by sex difference, but sufficiently large unbiased female samples for adipokine outcomes are not available to test this possibility. Finally, epigenetic phenomena that were independent of MR design, for example, methylation, acetylation, or potential mechanism of developmental compensation, also could affect the association of adipokine levels with PET risk.

## Conclusion

Our bidirectional MR study did not support a critical role for increased circulating adipokine levels in PET but identified a potential causality of PET risk and circulating PAI-1 levels, suggesting that PAI-1 may be a potential biomarker for the diagnosis or therapy of PET. Subsequent studies with updated data from large GWAS datasets are still required to authenticate the mentioned findings.

## Data Availability

The original contributions presented in the study are included in the article/Supplementary Material; further inquiries can be directed to the corresponding author.
