# Supplementary material for: Circulating adipokine levels and preeclampsia: A bidirectional Mendelian randomization study
Source: Front Genet. 2022 Aug 22;13:935757. doi: 10.3389/fgene.2022.935757 (PMC9444139; doi:10.3389/fgene.2022.935757)
Supplement: Supplementary file 1 [file Table1.DOCX]

Supplementary Table 1. Power calculation for Mendelian randomization analyses.

| Exposure | Outcome | Sample size | Type-I error rate | SNPs | R^2^ | Power (%) |
| --- | --- | --- | --- | --- | --- | --- |
| Forward |  |  |  |  |  |  |
| Adiponectin | PET | 39883 | 0.05 | 11 | 0.060 | 70.6 |
| Leptin | PET | 32161 | 0.05 | 4 | 0.017 | 23.3 |
| Resistin | PET | 30931 | 0.05 | 10 | 0.047 | 43.4 |
| sOB-R | PET | 1338 | 0.05 | 3 | 0.090 | 3.9 |
| PAI-1 | PET | 30395 | 0.05 | 4 | 0.019 | 81.8 |
| Reverse |  |  |  |  |  |  |
| PET | Adiponectin | 141068 | 0.05 | 10 | 0.002 | 2.8 |
| PET | Leptin | 141068 | 0.05 | 7 | 0.001 | 3.3 |
| PET | Resistin | 141068 | 0.05 | 6 | 0.001 | 3.6 |
| PET | sOB-R | 141068 | 0.05 | 17 | 0.004 | 2.7 |
| PET | PAI-1 | 141068 | 0.05 | 17 | 0.004 | 26.2 |

R^2^, variance in exposure explained by the SNPs;

Power, calculated using an online calculator(https://sb452.shinyapps.io/power);

Abbreviation: PET, preeclampsia or eclampsia; SNP, single-nucleotide polymorphism.
