# Supplementary figures and images for: Circulating adipokine levels and preeclampsia: A bidirectional Mendelian randomization study
Source: Front Genet. 2022 Aug 22;13:935757. doi: 10.3389/fgene.2022.935757 (PMC9444139; doi:10.3389/fgene.2022.935757)

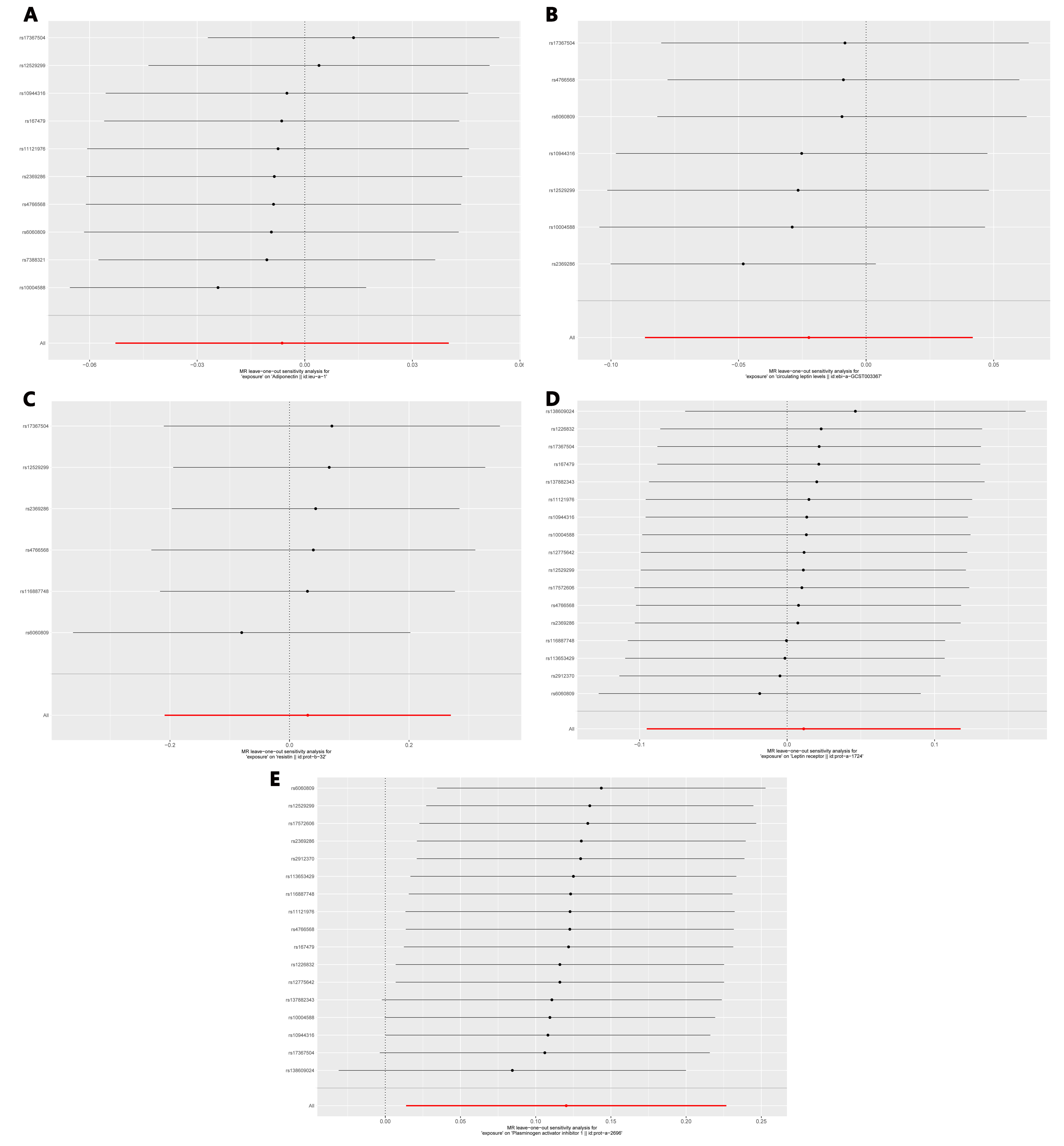

Supplement: Supplementary file 2 [file Image2.TIF]

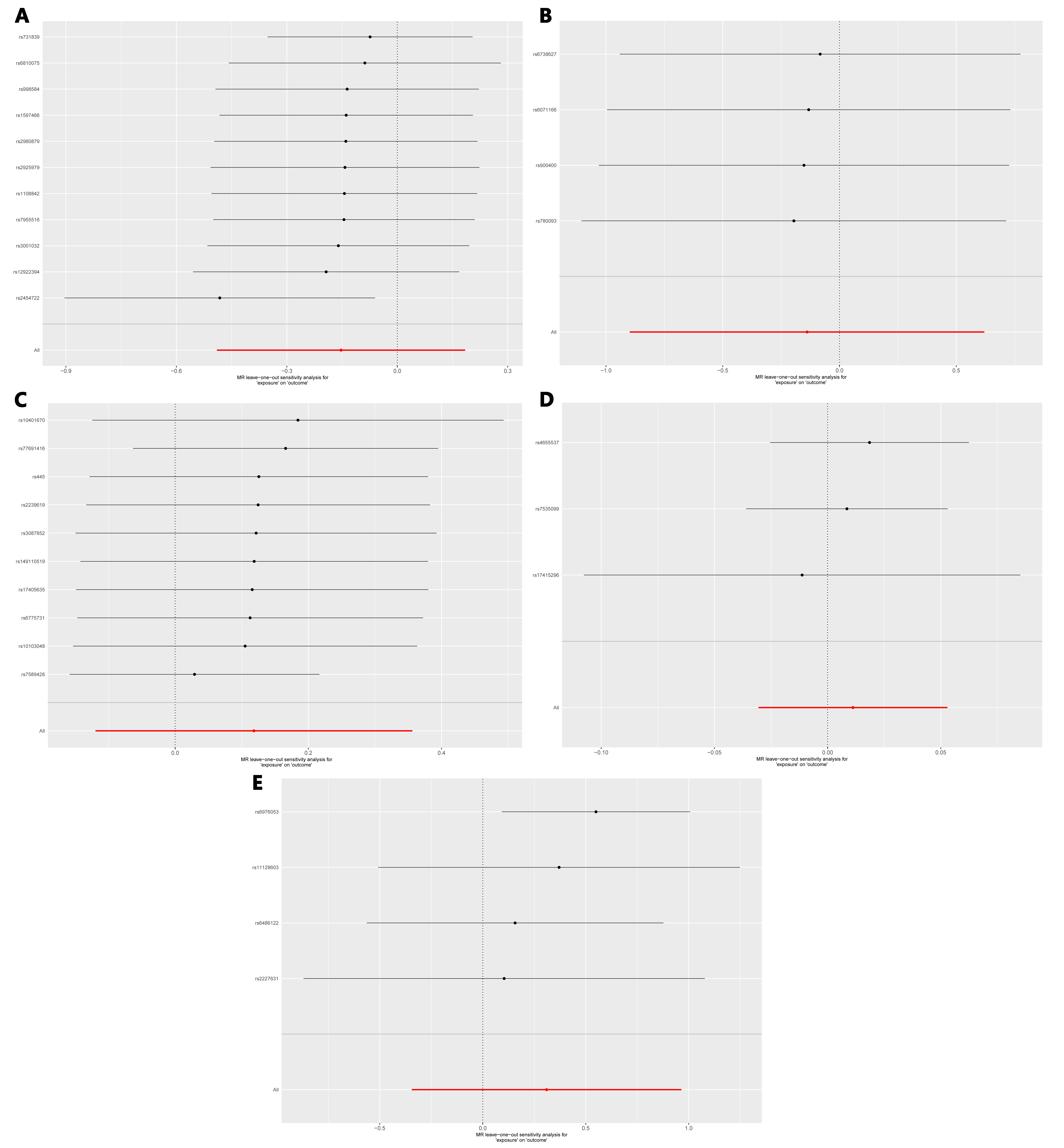

Supplement: Supplementary file 3 [file Image1.TIF]
