# Supplementary material for: Circulating adipokine levels and preeclampsia: A bidirectional Mendelian randomization study
Source: Front Genet. 2022 Aug 22;13:935757. doi: 10.3389/fgene.2022.935757 (PMC9444139; doi:10.3389/fgene.2022.935757)
Supplement: Supplementary file 4 [file Table2.DOCX]

Supplementary Table 2. Summary information on the SNPs used as genetic instruments for the five adipokines in the present MR study.

| SNPID | Chr | Position | Nearest Gene | EA | NEA | EAF | Adipokines | | | PET | | |
| --- | --- | --- | --- | --- | --- | --- | --- | --- | --- | --- | --- | --- |
| Adiponectin(exposure) |  |  |  |  |  |  | Beta | SE | P value | Beta | SE | P value |
| rs3001032 | 1 | 217794402 | LYPLAL1 | T | C | 0.7 | -0.02 | 0.004 | 3.60E-08 | -0.00666 | 0.02298 | 0.772 |
| rs1108842 | 3 | 52695120 | GNL3 | C | A | 0.5 | 0.03 | 0.004 | 1.39E-13 | -0.01156 | 0.02151 | 0.591 |
| rs1597466 | 3 | 151538251 | TSC22D2 | T | G | 0.1 | -0.03 | 0.007 | 1.62E-06 | 0.04963 | 0.04363 | 0.255 |
| rs6810075 | 3 | 188031259 | ADIPOQ | T | C | 0.6 | 0.06 | 0.004 | 1.19E-43 | -0.03125 | 0.02166 | 0.149 |
| rs998584 | 6 | 43865874 | VEGFA | C | A | 0.5 | 0.03 | 0.005 | 3.25E-08 | -0.01738 | 0.0216 | 0.421 |
| rs2980879 | 8 | 126550657 | TRIB1 | T | A | 0.7 | 0.03 | 0.005 | 7.13E-09 | -0.0171 | 0.02404 | 0.477 |
| rs7955516 | 12 | 20389303 | PDE3A | C | A | 0.4 | 0.02 | 0.004 | 4.45E-08 | -0.01419 | 0.02353 | 0.546 |
| rs2925979 | 16 | 80092291 | CMIP | T | C | 0.3 | -0.04 | 0.005 | 1.21E-20 | 0.01292 | 0.02302 | 0.575 |
| rs12922394 | 16 | 81229828 | CDH13 | T | C | 0.1 | -0.08 | 0.01 | 1.99E-15 | -0.02115 | 0.03757 | 0.574 |
| rs731839 | 19 | 38590905 | PEPD | G | A | 0.35 | -0.03 | 0.004 | 7.97E-12 | 0.07125 | 0.02261 | 0.00162 |
| rs2454722 | 12 | 123171218 | GPR109A | G | A | 0.166 | -0.15 | 0.03 | 3.87E-11 | -0.02172 | 0.02888 | 0.452 |
| Leptin(exposure) |  |  |  |  |  |  |  |  |  |  |  |  |
| rs780093 | 2 | 27596107 | GCKR | C | T | 0.61 | 0.032 | 0.005 | 2.33E-10 | -0.00028 | 0.02253 | 0.99 |
| rs900400 | 3 | 158281469 | CCNL1 | T | C | 0.6 | 0.03 | 0.005 | 5.60E-09 | -0.00299 | 0.02309 | 0.897 |
| rs6071166 | 20 | 36766426 | SLC32A1 | C | A | 0.37 | 0.027 | 0.006 | 6.56E-07 | -0.00439 | 0.02195 | 0.841 |
| rs6738627 | 2 | 165252696 | COBLL1 | A | G | 0.37 | 0.027 | 0.006 | 1.41E-06 | -0.00922 | 0.02246 | 0.681 |
| Resistin(exposure) |  |  |  |  |  |  |  |  |  |  |  |  |
| rs3087852 | 17 | 38137364 | PSMD3 | A | G | 0.46 | -0.086 | 0.0094 | 6.60E-20 | -0.0083 | 0.02165 | 0.702 |
| rs6775731 | 3 | 128306894 | RPN1 | T | C | 0.3 | -0.063 | 0.011 | 3.20E-09 | -0.01316 | 0.02395 | 0.583 |
| rs10103048 | 8 | 130602281 | GSDMC | A | C | 0.42 | 0.06 | 0.0096 | 5.20E-10 | 0.01866 | 0.02189 | 0.394 |
| rs17405635 | 2 | 43355763 | ZFP36L2 | A | G | 0.26 | 0.08 | 0.011 | 6.60E-14 | 0.01151 | 0.02458 | 0.64 |
| rs2239619 | 6 | 52453220 | TRAM2 | A | C | 0.62 | -0.053 | 0.0097 | 4.10E-08 | 0.00017 | 0.02189 | 0.994 |
| rs73008259 | 6 | 144411338 | SF3B5 | A | G | 0.053 | 0.19 | 0.02 | 3.70E-21 | 0.02133 | 0.06906 | 0.757 |
| rs445 | 7 | 92408370 | CDK6 | T | C | 0.1 | -0.085 | 0.015 | 3.30E-08 | 0.01143 | 0.04647 | 0.806 |
| rs7589428 | 2 | 43561771 | THADA | A | G | 0.51 | 0.063 | 0.0095 | 4.60E-11 | 0.07892 | 0.02151 | 0.000243 |
| rs77691416 | 6 | 144354119 | PLAGL1 | A | C | 0.91 | 0.12 | 0.016 | 7.90E-14 | -0.0634 | 0.04222 | 0.133 |
| rs10401670 | 19 | 7742802 | MCEMP1 | T | C | 0.43 | 0.15 | 0.012 | 9.00E-37 | 8.00E-04 | 0.02274 | 0.972 |
| sOB-R(exposure) |  |  |  |  |  |  |  |  |  |  |  |  |
| rs17415296 | 1 | 66099013 | LEPR | C | A | 0.17 | 0.14 | 0.033 | 4.47E-229 | 0.0229 | 0.03306 | 0.488 |
| rs4655537 | 1 | 66058801 | LEPR | A | G | 0.36 | 0.35 | 0.044 | 7.42E-15 | -0.02007 | 0.02396 | 0.402 |
| rs7535099 | 1 | 66050997 | LEPR | G | A | 0.24 | 0.346 | 0.051 | 3.06E-11 | 0.01311 | 0.02446 | 0.592 |
| PAI-1(exposure) |  |  |  |  |  |  |  |  |  |  |  |  |
| rs11128603 | 3 | 12385828 | PPARG | A | G | 0.14 | 0.066 | 0.012 | 2.90E-08 | 0.00473 | 0.02858 | 0.869 |
| rs2227631 | 7 | 100769538 | SERPINE1 | A | G | 0.44 | 0.073 | 0.007 | 3.20E-24 | 0.04219 | 0.02161 | 0.0508 |
| rs6976053 | 7 | 100512119 | ACHE | T | C | 0.17 | 0.048 | 0.007 | 5.80E-13 | -0.03445 | 0.02153 | 0.11 |
| rs6486122 | 11 | 13361524 | ARNTL | T | C | 0.41 | 0.046 | 0.0072 | 1.70E-10 | 0.04778 | 0.02156 | 0.0267 |

Abbreviation: Chr, chromosome; EA, effect allele; NEA, non-effect allele; EAF, effect allele frequency; SE, standard error.
