# Supplementary material for: Circulating adipokine levels and preeclampsia: A bidirectional Mendelian randomization study
Source: Front Genet. 2022 Aug 22;13:935757. doi: 10.3389/fgene.2022.935757 (PMC9444139; doi:10.3389/fgene.2022.935757)
Supplement: Supplementary file 5 [file Table5.DOCX]

Supplementary Table 5. Summary information on the SNPs used as genetic instruments for PET in the present MR study.

| SNPID | Chr | Position | Nearest Gene | EA | NEA | EAF | PET | | | Adipokines | | |
| --- | --- | --- | --- | --- | --- | --- | --- | --- | --- | --- | --- | --- |
| Adiponectin(outcome) |  |  |  |  |  |  | Beta | SE | P value | Beta | SE | P value |
| rs10004588 | 4 | 126412891 | FAT4 | A | C | 0.0452 | 0.24205 | 0.05247 | 3.97E-06 | 0.0255 | 0.010326 | 0.016659 |
| rs10944316 | 6 | 88261817 | RARS2 | T | C | 0.0695 | 0.20027 | 0.04298 | 3.17E-06 | -0.00594 | 0.01362 | 0.672218 |
| rs11121976 | 1 | 12833428 | PRAMEF12 | T | C | 0.162 | -0.14211 | 0.0292 | 1.13E-06 | -5.60E-05 | 0.005938 | 0.992716 |
| rs12529299 | 6 | 154942072 | CNKSR3 | T | C | 0.288 | -0.10856 | 0.02373 | 4.75E-06 | 0.009802 | 0.005207 | 0.067963 |
| rs167479 | 19 | 11526765 | RGL3 | G | T | 0.574 | 0.10815 | 0.02172 | 6.38E-07 | 0.00083 | 0.018735 | 0.965733 |
| rs17367504 | 1 | 11862778 | MTHFR | G | A | 0.145 | -0.14975 | 0.03066 | 1.03E-06 | 0.017163 | 0.00602 | 0.005716 |
| rs2369286 | 4 | 4595540 | STX18-AS1 | A | G | 0.255 | -0.1144 | 0.02474 | 3.75E-06 | -0.00108 | 0.005204 | 0.841215 |
| rs4766568 | 12 | 111725185 | CUX2 | C | T | 0.16 | -0.14111 | 0.02968 | 2.00E-06 | -0.00158 | 0.006457 | 0.812097 |
| rs6060809 | 20 | 34717350 | EPB41L1 | T | C | 0.0348 | 0.26984 | 0.05881 | 4.47E-06 | 0.004167 | 0.012302 | 0.742574 |
| rs7388321 | 8 | 17939143 | ASAH1 | C | G | 0.971 | -0.31406 | 0.06412 | 9.67E-07 | -0.04253 | 0.029159 | 0.15728 |
| Leptin(outcome) |  |  |  |  |  |  |  |  |  |  |  |  |
| rs10004588 | 4 | 126412891 | FAT4 | A | C | 0.0452 | 0.24205 | 0.05247 | 3.97E-06 | 0.0027 | 0.0148 | 0.854 |
| rs10944316 | 6 | 88261817 | RARS2 | T | C | 0.0695 | 0.20027 | 0.04298 | 3.17E-06 | 0.0021 | 0.0176 | 0.9051 |
| rs12529299 | 6 | 154942072 | CNKSR3 | T | C | 0.288 | -0.10856 | 0.02373 | 4.75E-06 | -6.00E-04 | 0.0074 | 0.9364 |
| rs17367504 | 1 | 11862778 | MTHFR | G | A | 0.145 | -0.14975 | 0.03066 | 1.03E-06 | 0.013 | 0.0087 | 0.135 |
| rs2369286 | 4 | 4595540 | STX18-AS1 | A | G | 0.255 | -0.1144 | 0.02474 | 3.75E-06 | -0.0164 | 0.0077 | 0.03313 |
| rs4766568 | 12 | 111725185 | CUX2 | C | T | 0.16 | -0.14111 | 0.02968 | 2.00E-06 | 0.0149 | 0.0093 | 0.1088 |
| rs6060809 | 20 | 34717350 | EPB41L1 | T | C | 0.0348 | 0.26984 | 0.05881 | 4.47E-06 | -0.0228 | 0.016 | 0.154 |
| Resistin(outcome) |  |  |  |  |  |  |  |  |  |  |  |  |
| rs116887748 | 18 | 60358234 | PHLPP1 | T | C | 0.0375 | 0.25939 | 0.05674 | 4.85E-06 | 0.0099 | 0.1321 | 0.9404 |
| rs12529299 | 6 | 154941570 | CNKSR3 | T | C | 0.288 | -0.10856 | 0.02373 | 4.75E-06 | 0.0174 | 0.0333 | 0.6004 |
| rs17367504 | 1 | 11862778 | MTHFR | G | A | 0.145 | -0.14975 | 0.03066 | 1.03E-06 | 0.0114 | 0.0349 | 0.7428 |
| rs2369286 | 4 | 4595540 | STX18-AS1 | A | G | 0.255 | -0.1144 | 0.02474 | 3.75E-06 | 0.1537 | 0.143 | 0.284 |
| rs4766568 | 12 | 111724699 | CUX2 | C | T | 0.16 | -0.14111 | 0.02968 | 2.00E-06 | 4.00E-04 | 0.0368 | 0.9909 |
| rs6060809 | 20 | 34717350 | EPB41L1 | T | C | 0.0348 | 0.26984 | 0.05881 | 4.47E-06 | 0.0849 | 0.0623 | 0.173 |
| sOB-R(outcome) |  |  |  |  |  |  |  |  |  |  |  |  |
| rs10004588 | 4 | 126390454 | FAT4 | A | C | 0.0452 | 0.24205 | 0.05247 | 3.97E-06 | -0.004 | 0.0532 | 0.933254 |
| rs10944316 | 6 | 88261817 | RARS2 | T | C | 0.0695 | 0.20027 | 0.04298 | 3.17E-06 | -0.0123 | 0.0653 | 0.851138 |
| rs11121976 | 1 | 12833428 | PRAMEF12 | T | C | 0.162 | -0.14211 | 0.0292 | 1.13E-06 | 0.0073 | 0.0333 | 0.831764 |
| rs113653429 | 1 | 21557407 | ECE1 | C | T | 0.0336 | 0.27196 | 0.0595 | 4.86E-06 | 0.0995 | 0.0794 | 0.20893 |
| rs116887748 | 18 | 60358234 | PHLPP1 | T | C | 0.0375 | 0.25939 | 0.05674 | 4.85E-06 | 0.1447 | 0.0974 | 0.138038 |
| rs1226832 | 1 | 45595626 | ZSWIM5 | C | G | 0.127 | -0.15446 | 0.03252 | 2.04E-06 | 0.034 | 0.038 | 0.371535 |
| rs12529299 | 6 | 154941570 | CNKSR3 | T | C | 0.288 | -0.10856 | 0.02373 | 4.75E-06 | -0.0018 | 0.0289 | 0.954993 |
| rs12775642 | 10 | 121712667 | MIR4682 | A | G | 0.323 | 0.11353 | 0.02313 | 9.22E-07 | 7.00E-04 | 0.0279 | 0.977237 |
| rs137882343 | 18 | 65010976 | DSEL | T | G | 0.0112 | 0.51261 | 0.10619 | 1.38E-06 | -0.0301 | 0.083 | 0.724436 |
| rs138609024 | 17 | 32119136 | ASIC2 | C | T | 0.00627 | 0.73836 | 0.15008 | 8.67E-07 | -0.1389 | 0.1037 | 0.18197 |
| rs167479 | 19 | 11526765 | RGL3 | G | T | 0.574 | 0.10815 | 0.02172 | 6.38E-07 | -0.0181 | 0.0252 | 0.467735 |
| rs17367504 | 1 | 11862778 | MTHFR | G | A | 0.145 | -0.14975 | 0.03066 | 1.03E-06 | 0.0237 | 0.0337 | 0.47863 |
| rs17572606 | 22 | 24868172 | ADORA2A-AS1 | T | C | 0.00985 | 0.54056 | 0.11465 | 2.42E-06 | 0.0124 | 0.0949 | 0.891251 |
| rs2369286 | 4 | 4595540 | STX18-AS1 | A | G | 0.255 | -0.1144 | 0.02474 | 3.75E-06 | -0.0097 | 0.0274 | 0.724436 |
| rs2912370 | 15 | 39050654 | LOC102724253 | C | T | 0.521 | -0.09989 | 0.02171 | 4.19E-06 | -0.0353 | 0.0256 | 0.169824 |
| rs4766568 | 12 | 111724699 | CUX2 | C | T | 0.16 | -0.14111 | 0.02968 | 2.00E-06 | -0.0117 | 0.0355 | 0.74131 |
| rs6060809 | 20 | 34717350 | EPB41L1 | T | C | 0.0348 | 0.26984 | 0.05881 | 4.47E-06 | 0.1571 | 0.0658 | 0.016982 |
| PAI-1(outcome) |  |  |  |  |  |  |  |  |  |  |  |  |
| rs10004588 | 4 | 126390454 | FAT4 | A | C | 0.0452 | 0.24205 | 0.05247 | 3.97E-06 | 0.0695 | 0.0532 | 0.190546 |
| rs10944316 | 6 | 88261817 | RARS2 | T | C | 0.0695 | 0.20027 | 0.04298 | 3.17E-06 | 0.1094 | 0.0653 | 0.093325 |
| rs11121976 | 1 | 12833428 | PRAMEF12 | T | C | 0.162 | -0.14211 | 0.0292 | 1.13E-06 | -0.0107 | 0.0334 | 0.74131 |
| rs113653429 | 1 | 21557407 | ECE1 | C | T | 0.0336 | 0.27196 | 0.0595 | 4.86E-06 | -0.0034 | 0.0794 | 0.977237 |
| rs116887748 | 18 | 60358234 | PHLPP1 | T | C | 0.0375 | 0.25939 | 0.05674 | 4.85E-06 | -0.0042 | 0.0974 | 0.954993 |
| rs1226832 | 1 | 45595626 | ZSWIM5 | C | G | 0.127 | -0.15446 | 0.03252 | 2.04E-06 | -0.0313 | 0.038 | 0.40738 |
| rs12529299 | 6 | 154941570 | CNKSR3 | T | C | 0.288 | -0.10856 | 0.02373 | 4.75E-06 | 0.0261 | 0.0289 | 0.363078 |
| rs12775642 | 10 | 121712667 | MIR4682 | A | G | 0.323 | 0.11353 | 0.02313 | 9.22E-07 | 0.023 | 0.0279 | 0.40738 |
| rs137882343 | 18 | 65010976 | DSEL | T | G | 0.0112 | 0.51261 | 0.10619 | 1.38E-06 | 0.1003 | 0.083 | 0.229087 |
| rs138609024 | 17 | 32119136 | ASIC2 | C | T | 0.00627 | 0.73836 | 0.15008 | 8.67E-07 | 0.2389 | 0.1036 | 0.02138 |
| rs167479 | 19 | 11526765 | RGL3 | G | T | 0.574 | 0.10815 | 0.02172 | 6.38E-07 | 0.0101 | 0.0252 | 0.691831 |
| rs17367504 | 1 | 11862778 | MTHFR | G | A | 0.145 | -0.14975 | 0.03066 | 1.03E-06 | -0.0525 | 0.0337 | 0.120226 |
| rs17572606 | 22 | 24868172 | ADORA2A-AS1 | T | C | 0.00985 | 0.54056 | 0.11465 | 2.42E-06 | -0.0082 | 0.0949 | 0.933254 |
| rs2369286 | 4 | 4595540 | STX18-AS1 | A | G | 0.255 | -0.1144 | 0.02474 | 3.75E-06 | 0.0074 | 0.0274 | 0.794328 |
| rs2912370 | 15 | 39050654 | LOC102724253 | C | T | 0.521 | -0.09989 | 0.02171 | 4.19E-06 | 0.0083 | 0.0256 | 0.74131 |
| rs4766568 | 12 | 111724699 | CUX2 | C | T | 0.16 | -0.14111 | 0.02968 | 2.00E-06 | -0.0101 | 0.0355 | 0.776247 |
| rs6060809 | 20 | 34717350 | EPB41L1 | T | C | 0.0348 | 0.26984 | 0.05881 | 4.47E-06 | -0.0885 | 0.0659 | 0.177828 |

Abbreviation: Chr, chromosome; EA, effect allele; NEA, non-effect allele; EAF, effect allele frequency; SE, standard error.
