# Supplementary material for: Circulating adipokine levels and preeclampsia: A bidirectional Mendelian randomization study
Source: Front Genet. 2022 Aug 22;13:935757. doi: 10.3389/fgene.2022.935757 (PMC9444139; doi:10.3389/fgene.2022.935757)
Supplement: Supplementary file 6 [file Table3.DOCX]

Supplementary Table 3. ORs (95% CIs) for MVMR Associations of five adipokine levels with PET.

| Exposure | Outcome | IVW OR | 95% CI | P value |
| --- | --- | --- | --- | --- |
| Adiponectin | PET | 0.99 | 0.73-1.35 | 0.956 |
| Leptin | PET | 0.9 | 0.45-1.82 | 0.778 |
| Resistin | PET | 1.03 | 0.80-1.31 | 0.843 |
| sOB-R | PET | 1.01 | 0.94-1.09 | 0.699 |
| PAI-1 | PET | 0.98 | 0.81-1.19 | 0.838 |

Abbreviation: MVMR, multi-variable Mendelian randomization; OR, odds ratio; IVW, inverse-variance weighted; 95% CI, 95% confidence interval.
