# Supplementary material for: Circulating adipokine levels and preeclampsia: A bidirectional Mendelian randomization study
Source: Front Genet. 2022 Aug 22;13:935757. doi: 10.3389/fgene.2022.935757 (PMC9444139; doi:10.3389/fgene.2022.935757)
Supplement: Supplementary file 7 [file Table4.DOCX]

Supplementary Table 4. Traits associated with adipokines-associated SNPs in PhenoScanner V2 at the genome-wide significance level.

| Adipokine | SNPID | Chr | | Nearest Gene | | EA | | Associated traits/diseases | | Beta | | P value | |  |
| --- | --- | --- | --- | --- | --- | --- | --- | --- | --- | --- | --- | --- | --- | --- |
| Adiponectin | rs3001032 | | 1 | | LYPLAL1 | | T | | Waist hip ratio | | -0.0411 | | 9.84E-15 | |
|  |  | |  | |  | |  | | Arm fat mass left | | 0.01497 | | 4.25E-09 | |
|  |  | |  | |  | |  | | Arm fat mass right | | 0.01538 | | 1.60E-09 | |
|  |  | |  | |  | |  | | Arm fat percentage left | | 0.01624 | | 1.62E-16 | |
|  |  | |  | |  | |  | | Arm fat percentage right | | 0.01631 | | 1.61E-16 | |
|  |  | |  | |  | |  | | Body fat percentage | | 0.01872 | | 6.21E-21 | |
|  |  | |  | |  | |  | | Hip circumference | | 0.02821 | | 6.26E-28 | |
|  |  | |  | |  | |  | | Impedance of arm left | | 0.02158 | | 7.08E-32 | |
|  |  | |  | |  | |  | | Impedance of arm right | | 0.02173 | | 1.44E-32 | |
|  |  | |  | |  | |  | | Impedance of whole body | | 0.0183 | | 2.69E-20 | |
|  |  | |  | |  | |  | | Leg fat percentage left | | 0.009434 | | 6.59E-09 | |
|  |  | |  | |  | |  | | Leg fat percentage right | | 0.009809 | | 2.60E-09 | |
|  |  | |  | |  | |  | | Trunk fat mass | | 0.02258 | | 6.60E-18 | |
|  |  | |  | |  | |  | | Trunk fat percentage | | 0.0253 | | 3.45E-26 | |
|  |  | |  | |  | |  | | Whole body fat mass | | 0.01867 | | 1.86E-13 | |
|  | rs1108842 | | 3 | | GNL3 | | C | | Body mass index | | -0.0222 | | 5.19E-10 | |
|  |  | |  | |  | |  | | Hematocrit | | 0.01947 | | 3.22E-08 | |
|  |  | |  | |  | |  | | High light scatter percentage of red cells | | 0.02331 | | 6.93E-11 | |
|  |  | |  | |  | |  | | High light scatter reticulocyte count | | 0.02614 | | 2.60E-13 | |
|  |  | |  | |  | |  | | Red blood cell count | | 0.01974 | | 2.61E-08 | |
|  |  | |  | |  | |  | | Reticulocyte count | | 0.02796 | | 5.79E-15 | |
|  |  | |  | |  | |  | | Reticulocyte fraction of red cells | | 0.02381 | | 2.87E-11 | |
|  |  | |  | |  | |  | | Waist hip ratio | | 0.022 | | 1.80E-10 | |
|  |  | |  | |  | |  | | Waist circumference | | 0.0256 | | 2.15E-09 | |
|  |  | |  | |  | |  | | Intelligence multi trait analysis | | 0.01682 | | 8.00E-09 | |
|  |  | |  | |  | |  | | Age at menarche | | -0.01442 | | 5.86E-09 | |
|  |  | |  | |  | |  | | Impedance of arm left | | 0.01449 | | 2.96E-17 | |
|  |  | |  | |  | |  | | Impedance of arm right | | 0.0141 | | 1.49E-16 | |
|  |  | |  | |  | |  | | Impedance of leg left | | 0.01788 | | 6.78E-16 | |
|  |  | |  | |  | |  | | Impedance of leg right | | 0.01645 | | 7.01E-14 | |
|  |  | |  | |  | |  | | Impedance of whole body | | 0.01762 | | 1.87E-21 | |
|  |  | |  | |  | |  | | Cross disorder | | 0.07139 | | 3.54E-09 | |
|  |  | |  | |  | |  | | Schizophrenia | | 0.0632 | | 2.27E-09 | |
|  | rs1597466 | | 3 | | TSC22D2 | | T | | Impedance of arm left | | 0.01713 | | 6.57E-09 | |
|  |  | |  | |  | |  | | Impedance of arm right | | 0.0165 | | 2.01E-08 | |
|  |  | |  | |  | |  | | Impedance of whole body | | 0.01742 | | 4.68E-08 | |
|  | rs6810075 | | 3 | | ADIPOQ | | T | | NA | | NA | | NA | |
|  | rs998584 | | 6 | | VEGFA | | C | | Granulocyte count | | -0.0204 | | 1.37E-08 | |
|  |  | |  | |  | |  | | Hemoglobin concentration | | -0.02031 | | 1.06E-08 | |
|  |  | |  | |  | |  | | High light scatter percentage of red cells | | -0.02321 | | 9.87E-11 | |
|  |  | |  | |  | |  | | High light scatter reticulocyte count | | -0.02582 | | 6.24E-13 | |
|  |  | |  | |  | |  | | Myeloid white cell count | | -0.02084 | | 7.18E-09 | |
|  |  | |  | |  | |  | | Neutrophil count | | -0.02041 | | 1.25E-08 | |
|  |  | |  | |  | |  | | Reticulocyte count | | -0.0263 | | 2.52E-13 | |
|  |  | |  | |  | |  | | Reticulocyte fraction of red cells | | -0.02319 | | 1.09E-10 | |
|  |  | |  | |  | |  | | Sum basophil neutrophil counts | | -0.02039 | | 1.36E-08 | |
|  |  | |  | |  | |  | | Sum neutrophil eosinophil counts | | -0.02046 | | 1.18E-08 | |
|  |  | |  | |  | |  | | White blood cell count | | -0.0208 | | 6.62E-09 | |
|  |  | |  | |  | |  | | Body mass index | | 0.014 | | 7.90E-10 | |
|  |  | |  | |  | |  | | Waist circumference | | -0.0553 | | 3.93E-26 | |
|  |  | |  | |  | |  | | High density lipoprotein | | 0.026 | | 2.27E-11 | |
|  |  | |  | |  | |  | | Triglycerides | | -0.0293 | | 3.42E-15 | |
|  |  | |  | |  | |  | | HDL cholesterol | | -0.026 | | 2.00E-11 | |
|  |  | |  | |  | |  | | Triglycerides | | -0.49 | | 3.00E-15 | |
|  |  | |  | |  | |  | | Waist to hip ratio | | -0.0631 | | 7.00E-34 | |
|  |  | |  | |  | |  | | White blood cell count | | -0.0208 | | 7.00E-09 | |
|  |  | |  | |  | |  | | Arm fat percentage left | | 0.01067 | | 7.28E-09 | |
|  |  | |  | |  | |  | | Arm fat percentage right | | 0.01066 | | 8.71E-09 | |
|  |  | |  | |  | |  | | Hip circumference | | 0.02112 | | 2.03E-18 | |
|  |  | |  | |  | |  | | Self-reported high cholesterol | | -0.00441 | | 3.48E-08 | |
|  |  | |  | |  | |  | | Coronary artery disease | | -0.0332 | | 1.21E-09 | |
|  | rs2980879 | | 8 | | TRIB1 | | T | | Lymphocyte percentage of white cells | | -0.02394 | | 3.67E-10 | |
|  |  | |  | |  | |  | | Mean corpuscular hemoglobin | | -0.02562 | | 1.50E-11 | |
|  |  | |  | |  | |  | | Mean corpuscular hemoglobin concentration | | -0.03018 | | 4.27E-16 | |
|  |  | |  | |  | |  | | Neutrophil percentage of white cells | | 0.02462 | | 1.24E-10 | |
|  |  | |  | |  | |  | | Red blood cell count | | 0.02263 | | 2.96E-09 | |
|  |  | |  | |  | |  | | Red cell distribution width | | 0.04025 | | 2.75E-26 | |
|  |  | |  | |  | |  | | Reticulocyte fraction of red cells | | -0.02615 | | 1.08E-11 | |
|  |  | |  | |  | |  | | High density lipoprotein | | 0.0398 | | 1.63E-23 | |
|  |  | |  | |  | |  | | Low density lipoprotein | | -0.0358 | | 2.96E-18 | |
|  |  | |  | |  | |  | | Total cholesterol | | -0.0402 | | 2.42E-25 | |
|  |  | |  | |  | |  | | Triglycerides | | -0.0637 | | 8.02E-67 | |
|  |  | |  | |  | |  | | HDL cholesterol | | NA | | 2.93E-13 | |
|  |  | |  | |  | |  | | LDL cholesterol | | NA | | 3.12E-12 | |
|  |  | |  | |  | |  | | Arm fat percentage left | | 0.01098 | | 2.95E-08 | |
|  |  | |  | |  | |  | | Arm fat percentage right | | 0.01089 | | 4.39E-08 | |
|  |  | |  | |  | |  | | Self-reported high cholesterol | | -0.01098 | | 1.45E-37 | |
|  |  | |  | |  | |  | | Treatment with atorvastatin | | -0.00265 | | 3.31E-09 | |
|  |  | |  | |  | |  | | Treatment with cholesterol lowering medication | | -0.00905 | | 3.42E-14 | |
|  |  | |  | |  | |  | | Treatment with simvastatin | | -0.00793 | | 1.16E-21 | |
|  |  | |  | |  | |  | | Trunk fat percentage | | 0.01341 | | 2.43E-08 | |
|  |  | |  | |  | |  | | Coronary artery disease | | -0.0444 | | 3.31E-16 | |
|  | rs7955516 | | 12 | | PDE3A | | C | | Pulse rate | | 0.01741 | | 1.20E-11 | |
|  | rs2925979 | | 16 | | CMIP | | T | | Waist circumference | | 0.0357 | | 1.01E-10 | |
|  |  | |  | |  | |  | | High density lipoprotein | | -0.0351 | | 1.32E-19 | |
|  |  | |  | |  | |  | | HDL cholesterol levels | | 0.0351 | | 9.00E-22 | |
|  |  | |  | |  | |  | | Type 2 diabetes | | 0.06766 | | 2.00E-09 | |
|  |  | |  | |  | |  | | Waist to hip ratio | | 0.0342 | | 7.00E-13 | |
|  |  | |  | |  | |  | | Impedance of arm left | | -0.0122 | | 6.70E-11 | |
|  |  | |  | |  | |  | | Impedance of arm right | | -0.01311 | | 1.86E-12 | |
|  |  | |  | |  | |  | | Self-reported hypertension | | 0.006622 | | 1.18E-08 | |
|  |  | |  | |  | |  | | Treatment with cholesterol lowering medication | | 0.007207 | | 2.19E-09 | |
|  |  | |  | |  | |  | | Vascular or heart problems diagnosed by doctor | | -0.00768 | | 2.08E-10 | |
|  |  | |  | |  | |  | | Cholesterol hdl | | NA | | 2.00E-11 | |
|  | rs12922394 | | 16 | | CDH13 | | T | | NA | | NA | | NA | |
|  | rs731839 | | 19 | | PEPD | | G | | High density lipoprotein | | -0.022 | | 3.44E-09 | |
|  |  | |  | |  | |  | | Triglycerides | | 0.0224 | | 2.65E-09 | |
|  |  | |  | |  | |  | | Body mass index | | NA | | 5.13E-12 | |
|  |  | |  | |  | |  | | Fasting insulin | | NA | | 5.10E-12 | |
|  |  | |  | |  | |  | | HDL cholesterol | | 0.022 | | 3.00E-09 | |
|  |  | |  | |  | |  | | Arm fat percentage left | | -0.01385 | | 1.25E-12 | |
|  |  | |  | |  | |  | | Arm fat percentage right | | -0.01409 | | 6.30E-13 | |
|  |  | |  | |  | |  | | Body fat percentage | | -0.01553 | | 3.72E-15 | |
|  |  | |  | |  | |  | | Impedance of arm left | | -0.016 | | 1.43E-18 | |
|  |  | |  | |  | |  | | Impedance of arm right | | -0.01572 | | 4.03E-18 | |
|  |  | |  | |  | |  | | Impedance of whole body | | -0.01382 | | 1.96E-12 | |
|  |  | |  | |  | |  | | Trunk fat mass | | -0.01924 | | 1.19E-13 | |
|  |  | |  | |  | |  | | Trunk fat percentage | | -0.02158 | | 8.04E-20 | |
|  |  | |  | |  | |  | | Whole body fat mass | | -0.01511 | | 1.85E-09 | |
|  | rs2454722 | | 12 | | GPR109A | | G | | Mean platelet volume | | -0.0481 | | 9.37E-25 | |
|  |  | |  | |  | |  | | High density lipoprotein | | -0.0351 | | 3.31E-14 | |
| Leptin | rs780093 | | 2 | | GCKR | | C | | Age at menopause | | 0.12 | | 2.30E-09 | |
|  |  | |  | |  | |  | | Alcohol intake frequency | | 0.04815 | | 1.21E-39 | |
|  |  | |  | |  | |  | | Arm fat-free mass left | | -0.01889 | | 1.29E-32 | |
|  |  | |  | |  | |  | | Arm fat-free mass right | | -0.01938 | | 1.35E-35 | |
|  |  | |  | |  | |  | | Arm predicted mass left | | -0.01868 | | 3.71E-32 | |
|  |  | |  | |  | |  | | Arm predicted mass right | | -0.01924 | | 2.43E-35 | |
|  |  | |  | |  | |  | | Basal metabolic rate | | -0.019 | | 1.79E-30 | |
|  |  | |  | |  | |  | | Blood glucose in non diabetics | | NA | | 2.80E-08 | |
|  |  | |  | |  | |  | | Comparative height size at age 10 | | -0.0144 | | 3.15E-17 | |
|  |  | |  | |  | |  | | Crohns disease | | NA | | 4.70E-11 | |
|  |  | |  | |  | |  | | Daytime dozing or sleeping | | 0.008019 | | 8.62E-11 | |
|  |  | |  | |  | |  | | Diabetes diagnosed by doctor | | -0.00374 | | 2.87E-12 | |
|  |  | |  | |  | |  | | DPA 22:5n3 | | 0.0163 | | 2.04E-08 | |
|  |  | |  | |  | |  | | Fasting glucose | | -0.028 | | 2.40E-38 | |
|  |  | |  | |  | |  | | Glomerular filtration rate creatinine | | 0.0081 | | 2.00E-16 | |
|  |  | |  | |  | |  | | Granulocyte count | | 0.02822 | | 1.63E-14 | |
|  |  | |  | |  | |  | | Granulocyte percentage of myeloid white cells | | 0.03947 | | 3.59E-27 | |
|  |  | |  | |  | |  | | Height | | -0.01607 | | 1.39E-19 | |
|  |  | |  | |  | |  | | Hematocrit | | -0.02158 | | 2.35E-09 | |
|  |  | |  | |  | |  | | High light scatter percentage of red cells | | 0.03423 | | 1.07E-20 | |
|  |  | |  | |  | |  | | High light scatter reticulocyte count | | 0.03113 | | 2.17E-17 | |
|  |  | |  | |  | |  | | Impedance of arm left | | 0.01877 | | 1.96E-26 | |
|  |  | |  | |  | |  | | Impedance of arm right | | 0.01856 | | 4.31E-26 | |
|  |  | |  | |  | |  | | Impedance of leg left | | 0.01874 | | 1.88E-16 | |
|  |  | |  | |  | |  | | Impedance of leg right | | 0.01832 | | 5.28E-16 | |
|  |  | |  | |  | |  | | Impedance of whole body | | 0.02035 | | 1.20E-26 | |
|  |  | |  | |  | |  | | Leg fat-free mass left | | -0.01715 | | 1.88E-25 | |
|  |  | |  | |  | |  | | Leg fat-free mass right | | -0.01712 | | 2.22E-25 | |
|  |  | |  | |  | |  | | Leg predicted mass left | | -0.01696 | | 3.08E-25 | |
|  |  | |  | |  | |  | | Leg predicted mass right | | -0.01706 | | 1.55E-25 | |
|  |  | |  | |  | |  | | log eGFR creatinine | | 0.0066 | | 8.90E-14 | |
|  |  | |  | |  | |  | | log Fasting insulin | | -0.018 | | 1.23E-13 | |
|  |  | |  | |  | |  | | Low density lipoprotein | | 0.0223 | | 2.36E-08 | |
|  |  | |  | |  | |  | | Lymphocyte count | | 0.0222 | | 1.69E-09 | |
|  |  | |  | |  | |  | | Metabolic syndrome x | | NA | | 2.00E-12 | |
|  |  | |  | |  | |  | | Monocyte percentage of white cells | | -0.04431 | | 5.10E-34 | |
|  |  | |  | |  | |  | | Myeloid white cell count | | 0.02509 | | 9.92E-12 | |
|  |  | |  | |  | |  | | Neutrophil count | | 0.02978 | | 4.48E-16 | |
|  |  | |  | |  | |  | | Palmitoleic acid 16:1n7 | | 0.0201 | | 9.80E-10 | |
|  |  | |  | |  | |  | | Plasma docosapentaenoic acid levels | | NA | | 2.04E-08 | |
|  |  | |  | |  | |  | | Plasma palmitoleic acid | | NA | | 9.80E-10 | |
|  |  | |  | |  | |  | | Plasma protein C levels | | NA | | 5.63E-17 | |
|  |  | |  | |  | |  | | Platelet count | | 0.03579 | | 8.21E-22 | |
|  |  | |  | |  | |  | | Plateletcrit | | 0.03368 | | 2.30E-19 | |
|  |  | |  | |  | |  | | Pulse rate | | 0.02776 | | 4.01E-27 | |
|  |  | |  | |  | |  | | Red blood cell count | | -0.02055 | | 1.59E-08 | |
|  |  | |  | |  | |  | | Red cell distribution width | | -0.02385 | | 4.65E-11 | |
|  |  | |  | |  | |  | | Reticulocyte count | | 0.03133 | | 1.55E-17 | |
|  |  | |  | |  | |  | | Reticulocyte fraction of red cells | | 0.03672 | | 1.58E-23 | |
|  |  | |  | |  | |  | | Self-reported diabetes | | -0.003 | | 6.65E-10 | |
|  |  | |  | |  | |  | | Self-reported gout | | 0.003213 | | 1.46E-27 | |
|  |  | |  | |  | |  | | Self-reported high cholesterol | | 0.009567 | | 1.55E-31 | |
|  |  | |  | |  | |  | | Serum creatinine | | NA | | 7.80E-11 | |
|  |  | |  | |  | |  | | Serum urate | | NA | | 3.65E-39 | |
|  |  | |  | |  | |  | | Sex hormone binding globulin levels | | 0.032 | | 2.00E-16 | |
|  |  | |  | |  | |  | | Sitting height | | -0.01811 | | 6.83E-21 | |
|  |  | |  | |  | |  | | Sodium in urine | | 0.01896 | | 6.43E-15 | |
|  |  | |  | |  | |  | | Sum basophil neutrophil counts | | 0.02918 | | 1.94E-15 | |
|  |  | |  | |  | |  | | Sum neutrophil eosinophil counts | | 0.02874 | | 4.76E-15 | |
|  |  | |  | |  | |  | | Total cholesterol levels | | 0.0515 | | 3.00E-47 | |
|  |  | |  | |  | |  | | Treatment with allopurinol | | 0.002678 | | 3.55E-24 | |
|  |  | |  | |  | |  | | Treatment with atorvastatin | | 0.00294 | | 6.36E-12 | |
|  |  | |  | |  | |  | | Treatment with cholesterol lowering medication | | 0.007894 | | 4.10E-12 | |
|  |  | |  | |  | |  | | Triglycerides | | 0.1106 | | 6.17E-220 | |
|  |  | |  | |  | |  | | Trunk fat-free mass | | -0.02082 | | 5.92E-40 | |
|  |  | |  | |  | |  | | Trunk predicted mass | | -0.02082 | | 3.20E-40 | |
|  |  | |  | |  | |  | | Type II diabetes | | -0.07974 | | 1.30E-09 | |
|  |  | |  | |  | |  | | Urate levels | | 5.15 | | 4.00E-17 | |
|  |  | |  | |  | |  | | Uric acid | | NA | | 4.00E-17 | |
|  |  | |  | |  | |  | | Waist circumference and Triglycerides | | NA | | 1.90E-12 | |
|  |  | |  | |  | |  | | Weight | | -0.01681 | | 1.39E-14 | |
|  |  | |  | |  | |  | | White blood cell count | | 0.02984 | | 4.11E-16 | |
|  |  | |  | |  | |  | | Whole body water mass | | -0.02019 | | 2.74E-37 | |
|  | rs900400 | | 3 | | CCNL1 | | T | | Age at menarche | | -0.034 | | 2.30E-11 | |
|  |  | |  | |  | |  | | Birth weight | | -0.04556 | | 4.03E-45 | |
|  |  | |  | |  | |  | | Heel bone mineral density | | -0.02849 | | 3.14E-19 | |
|  |  | |  | |  | |  | | Menarche age at onset | | -0.03 | | 2.00E-11 | |
|  |  | |  | |  | |  | | Newborn fat mass | | NA | | 8.55E-09 | |
|  |  | |  | |  | |  | | Newborn sum of skinfolds sqrt mm | | NA | | 4.08E-13 | |
|  |  | |  | |  | |  | | Ponderal index weightlength3 | | NA | | 9.50E-28 | |
|  |  | |  | |  | |  | | Ponderal index z score | | NA | | 5.00E-21 | |
|  |  | |  | |  | |  | | Sitting height | | 0.01436 | | 7.10E-14 | |
|  |  | |  | |  | |  | | Waist circumference | | -0.01598 | | 3.55E-13 | |
|  |  | |  | |  | |  | | Waist hip ratio | | -0.024 | | 3.80E-08 | |
|  | rs6071166 | | 20 | | SLC32A1 | | C | | NA | | NA | | NA | |
|  | rs6738627 | | 2 | | COBLL1 | | A | | Arm fat mass left | | 0.014 | | 1.81E-08 | |
|  |  | |  | |  | |  | | Arm fat mass right | | 0.01453 | | 5.06E-09 | |
|  |  | |  | |  | |  | | Arm fat percentage left | | 0.0157 | | 2.96E-16 | |
|  |  | |  | |  | |  | | Arm fat percentage right | | 0.01557 | | 7.00E-16 | |
|  |  | |  | |  | |  | | Body fat percentage | | 0.01763 | | 1.24E-19 | |
|  |  | |  | |  | |  | | Diabetes diagnosed by doctor | | -0.00314 | | 7.89E-09 | |
|  |  | |  | |  | |  | | High light scatter percentage of red cells | | -0.02104 | | 1.80E-08 | |
|  |  | |  | |  | |  | | High light scatter reticulocyte count | | -0.02308 | | 6.47E-10 | |
|  |  | |  | |  | |  | | Hip circumference | | 0.0235 | | 8.58E-21 | |
|  |  | |  | |  | |  | | Impedance of arm left | | 0.01455 | | 4.69E-16 | |
|  |  | |  | |  | |  | | Impedance of arm right | | 0.01425 | | 1.38E-15 | |
|  |  | |  | |  | |  | | Impedance of whole body | | 0.01236 | | 1.67E-10 | |
|  |  | |  | |  | |  | | Leg fat mass left | | 0.01117 | | 2.54E-08 | |
|  |  | |  | |  | |  | | Leg fat mass right | | 0.01178 | | 6.38E-09 | |
|  |  | |  | |  | |  | | Leg fat percentage left | | 0.01054 | | 3.05E-11 | |
|  |  | |  | |  | |  | | Leg fat percentage right | | 0.01135 | | 1.60E-12 | |
|  |  | |  | |  | |  | | Reticulocyte count | | -0.02714 | | 4.14E-13 | |
|  |  | |  | |  | |  | | Reticulocyte fraction of red cells | | -0.02434 | | 7.70E-11 | |
|  |  | |  | |  | |  | | Trunk fat mass | | 0.01932 | | 3.95E-14 | |
|  |  | |  | |  | |  | | Trunk fat percentage | | 0.02257 | | 3.69E-22 | |
|  |  | |  | |  | |  | | Type II diabetes | | -0.08618 | | 5.40E-11 | |
|  |  | |  | |  | |  | | Waist circumference | | -0.0483 | | 1.30E-22 | |
|  |  | |  | |  | |  | | Waist hip ratio | | -0.0495 | | 4.16E-19 | |
|  |  | |  | |  | |  | | Whole body fat mass | | 0.01666 | | 1.70E-11 | |
| Resistin | rs3087852 | | 17 | | PSMD3 | | A | | Allergic disease | | 0.0369 | | 1.24E-10 | |
|  |  | |  | |  | |  | | Basophil count | | 0.04736 | | 2.39E-41 | |
|  |  | |  | |  | |  | | Eosinophil percentage of granulocytes | | -0.03761 | | 1.35E-25 | |
|  |  | |  | |  | |  | | Eosinophil percentage of white cells | | -0.02265 | | 2.53E-10 | |
|  |  | |  | |  | |  | | Granulocyte count | | 0.1091 | | 6.47E-201 | |
|  |  | |  | |  | |  | | Granulocyte percentage of myeloid white cells | | 0.078 | | 1.03E-104 | |
|  |  | |  | |  | |  | | Lymphocyte percentage of white cells | | -0.07263 | | 1.46E-91 | |
|  |  | |  | |  | |  | | Monocyte count | | 0.01975 | | 3.76E-08 | |
|  |  | |  | |  | |  | | Monocyte percentage of white cells | | -0.05914 | | 2.64E-61 | |
|  |  | |  | |  | |  | | Myeloid white cell count | | 0.1062 | | 1.92E-189 | |
|  |  | |  | |  | |  | | Neutrophil count | | 0.1087 | | 1.88E-200 | |
|  |  | |  | |  | |  | | Neutrophil percentage of granulocytes | | 0.03472 | | 4.82E-22 | |
|  |  | |  | |  | |  | | Neutrophil percentage of white cells | | 0.08183 | | 2.47E-115 | |
|  |  | |  | |  | |  | | No blood clot, bronchitis, emphysema, asthma, rhinitis, eczema or allergy diagnosed by doctor | | -0.00847 | | 1.26E-13 | |
|  |  | |  | |  | |  | | Self-reported asthma | | 0.006323 | | 6.80E-16 | |
|  |  | |  | |  | |  | | Sum basophil neutrophil counts | | 0.1095 | | 9.73E-203 | |
|  |  | |  | |  | |  | | Sum eosinophil basophil counts | | 0.02072 | | 7.69E-09 | |
|  |  | |  | |  | |  | | Sum neutrophil eosinophil counts | | 0.1083 | | 8.20E-199 | |
|  |  | |  | |  | |  | | White blood cell count | | 0.09481 | | 9.86E-153 | |
|  | rs6775731 | | 3 | | RPN1 | | T | | Eosinophil count | | -0.04147 | | 1.12E-26 | |
|  |  | |  | |  | |  | | Eosinophil percentage of granulocytes | | -0.04831 | | 2.20E-35 | |
|  |  | |  | |  | |  | | Eosinophil percentage of white cells | | -0.04859 | | 4.47E-36 | |
|  |  | |  | |  | |  | | Granulocyte percentage of myeloid white cells | | -0.05757 | | 1.08E-49 | |
|  |  | |  | |  | |  | | Lymphocyte percentage of white cells | | -0.03066 | | 2.52E-15 | |
|  |  | |  | |  | |  | | Monocyte count | | 0.08067 | | 6.27E-96 | |
|  |  | |  | |  | |  | | Monocyte percentage of white cells | | 0.07571 | | 4.36E-85 | |
|  |  | |  | |  | |  | | Myeloid white cell count | | 0.02566 | | 5.65E-11 | |
|  |  | |  | |  | |  | | Neutrophil count | | 0.02149 | | 3.50E-08 | |
|  |  | |  | |  | |  | | Neutrophil percentage of granulocytes | | 0.04566 | | 8.85E-32 | |
|  |  | |  | |  | |  | | Sum eosinophil basophil counts | | -0.03817 | | 8.50E-23 | |
|  | rs10103048 | | 8 | | GSDMC | | A | | Basophil percentage of granulocytes | | -0.02035 | | 1.43E-08 | |
|  |  | |  | |  | |  | | Eosinophil percentage of granulocytes | | -0.03125 | | 1.60E-17 | |
|  |  | |  | |  | |  | | Eosinophil percentage of white cells | | -0.0297 | | 4.15E-16 | |
|  |  | |  | |  | |  | | Granulocyte count | | 0.03717 | | 5.56E-24 | |
|  |  | |  | |  | |  | | Granulocyte percentage of myeloid white cells | | -0.05896 | | 2.28E-58 | |
|  |  | |  | |  | |  | | Lymphocyte percentage of white cells | | -0.04048 | | 1.41E-28 | |
|  |  | |  | |  | |  | | Mean corpuscular hemoglobin | | 0.0241 | | 3.15E-11 | |
|  |  | |  | |  | |  | | Mean corpuscular volume | | 0.0299 | | 1.45E-16 | |
|  |  | |  | |  | |  | | Monocyte count | | 0.1021 | | 3.71E-171 | |
|  |  | |  | |  | |  | | Monocyte percentage of white cells | | 0.08143 | | 2.81E-110 | |
|  |  | |  | |  | |  | | Myeloid white cell count | | 0.04727 | | 1.46E-37 | |
|  |  | |  | |  | |  | | Neutrophil count | | 0.03915 | | 1.49E-26 | |
|  |  | |  | |  | |  | | Neutrophil percentage of granulocytes | | 0.03362 | | 4.86E-20 | |
|  |  | |  | |  | |  | | Neutrophil percentage of white cells | | 0.02236 | | 9.73E-10 | |
|  |  | |  | |  | |  | | Sum basophil neutrophil counts | | 0.03898 | | 3.00E-26 | |
|  |  | |  | |  | |  | | Sum neutrophil eosinophil counts | | 0.03737 | | 2.74E-24 | |
|  |  | |  | |  | |  | | White blood cell count | | 0.0389 | | 3.52E-26 | |
|  | rs17405635 | | 2 | | ZFP36L2 | | A | | Granulocyte percentage of myeloid white cells | | -0.03538 | | 4.22E-18 | |
|  |  | |  | |  | |  | | Monocyte count | | 0.02599 | | 1.83E-10 | |
|  |  | |  | |  | |  | | Monocyte percentage of white cells | | 0.03281 | | 7.28E-16 | |
|  | rs2239619 | | 6 | | TRAM2 | | A | | Total cholesterol levels | | -0.0186 | | 3.00E-08 | |
|  | rs73008259 | | 6 | | SF3B5 | | A | | Eosinophil percentage of granulocytes | | 0.04865 | | 1.24E-08 | |
|  |  | |  | |  | |  | | Granulocyte percentage of myeloid white cells | | -0.09298 | | 1.18E-27 | |
|  |  | |  | |  | |  | | Monocyte count | | 0.05872 | | 5.66E-12 | |
|  |  | |  | |  | |  | | Monocyte percentage of white cells | | 0.09128 | | 7.06E-27 | |
|  |  | |  | |  | |  | | Neutrophil count | | -0.04854 | | 1.37E-08 | |
|  |  | |  | |  | |  | | Neutrophil percentage of granulocytes | | -0.04866 | | 1.25E-08 | |
|  |  | |  | |  | |  | | Neutrophil percentage of white cells | | -0.06663 | | 5.14E-15 | |
|  |  | |  | |  | |  | | Sum basophil neutrophil counts | | -0.04927 | | 8.88E-09 | |
|  | rs445 | | 7 | | CDK6 | | T | | Basophil count | | 0.06863 | | 4.77E-31 | |
|  |  | |  | |  | |  | | Eosinophil count | | 0.05291 | | 1.84E-18 | |
|  |  | |  | |  | |  | | Granulocyte count | | -0.1038 | | 2.00E-65 | |
|  |  | |  | |  | |  | | Lymphocyte count | | NA | | 2.00E-08 | |
|  |  | |  | |  | |  | | Lymphocyte percentage of white cells | | -0.06534 | | 2.00E-27 | |
|  |  | |  | |  | |  | | Monocyte count | | 0.09809 | | 3.83E-59 | |
|  |  | |  | |  | |  | | Myeloid white cell count | | -0.1103 | | 3.00E-73 | |
|  |  | |  | |  | |  | | Neutrophil count | | -0.09798 | | 1.00E-58 | |
|  |  | |  | |  | |  | | Neutrophil percentage of white cells | | -0.04239 | | 2.00E-12 | |
|  |  | |  | |  | |  | | Neutrophils | | NA | | 7.00E-10 | |
|  |  | |  | |  | |  | | Sum basophil neutrophil counts | | -0.09926 | | 5.00E-60 | |
|  |  | |  | |  | |  | | Sum eosinophil basophil counts | | 0.0688 | | 4.85E-30 | |
|  |  | |  | |  | |  | | Sum neutrophil eosinophil counts | | 0.1026 | | 3.97E-64 | |
|  |  | |  | |  | |  | | White blood cell count | | 0.1037 | | 1.61E-65 | |
|  |  | |  | |  | |  | | White blood cell count basophil | | -0.06863 | | 5.00E-31 | |
|  |  | |  | |  | |  | | White blood cell types | | 0.076 | | 7.00E-10 | |
|  | rs7589428 | | 2 | | THADA | | A | | NA | | NA | | NA | |
|  | rs77691416 | | 6 | | PLAGL1 | | A | | NA | | NA | | NA | |
|  | rs10401670 | | 19 | | MCEMP1 | | T | | NA | | NA | | NA | |
| sOB-R | rs17415296 | | 1 | | LEPR | | C | | Blood protein levels | | 1.4 | | 4.00E-229 | |
|  | rs4655537 | | 1 | | LEPR | | A | | Blood protein levels | | 0.3502 | | 7.00E-15 | |
|  |  | |  | |  | |  | | C reactive protein | | NA | | 6.64E-10 | |
|  |  | |  | |  | |  | | Granulocyte count | | 0.03726 | | 3.16E-23 | |
|  |  | |  | |  | |  | | Granulocyte percentage of myeloid white cells | | 0.02056 | | 3.66E-08 | |
|  |  | |  | |  | |  | | Mean corpuscular hemoglobin | | -0.02092 | | 1.57E-08 | |
|  |  | |  | |  | |  | | Myeloid white cell count | | 0.03745 | | 2.51E-23 | |
|  |  | |  | |  | |  | | Neutrophil count | | 0.03691 | | 6.31E-23 | |
|  |  | |  | |  | |  | | Sum basophil neutrophil counts | | 0.0375 | | 1.57E-23 | |
|  |  | |  | |  | |  | | Sum neutrophil eosinophil counts | | 0.03683 | | 8.47E-23 | |
|  |  | |  | |  | |  | | White blood cell count | | 0.03678 | | 9.55E-23 | |
|  | rs7535099 | | 1 | | LEPR | | G | | Blood protein levels | | -0.3459 | | 3.00E-11 | |
| PAI-1 | rs11128603 | | 3 | | PPARG | | A | | Arm fat mass left | | -0.02308 | | 2.30E-10 | |
|  |  | |  | |  | |  | | Arm fat mass right | | -0.02359 | | 9.14E-11 | |
|  |  | |  | |  | |  | | Arm fat percentage left | | -0.02221 | | 2.80E-15 | |
|  |  | |  | |  | |  | | Arm fat percentage right | | -0.02353 | | 8.17E-17 | |
|  |  | |  | |  | |  | | Body fat percentage | | -0.02955 | | 3.19E-25 | |
|  |  | |  | |  | |  | | Impedance of arm left | | -0.02059 | | 4.22E-15 | |
|  |  | |  | |  | |  | | Impedance of arm right | | -0.02347 | | 2.63E-19 | |
|  |  | |  | |  | |  | | Impedance of leg left | | -0.02555 | | 4.58E-14 | |
|  |  | |  | |  | |  | | Impedance of leg right | | -0.02669 | | 2.00E-15 | |
|  |  | |  | |  | |  | | Impedance of whole body | | -0.02326 | | 2.20E-16 | |
|  |  | |  | |  | |  | | Leg fat mass left | | -0.02216 | | 4.40E-14 | |
|  |  | |  | |  | |  | | Leg fat mass right | | -0.02286 | | 1.39E-14 | |
|  |  | |  | |  | |  | | Leg fat percentage left | | -0.02097 | | 1.72E-19 | |
|  |  | |  | |  | |  | | Leg fat percentage right | | -0.02227 | | 2.92E-21 | |
|  |  | |  | |  | |  | | Trunk fat mass | | -0.03352 | | 3.17E-19 | |
|  |  | |  | |  | |  | | Trunk fat percentage | | -0.03648 | | 1.19E-26 | |
|  |  | |  | |  | |  | | Type II diabetes | | 0.12 | | 6.60E-11 | |
|  |  | |  | |  | |  | | Whole body fat mass | | -0.02948 | | 4.13E-16 | |
|  | rs2227631 | | 7 | | SERPINE1 | | A | | NA | | NA | | NA | |
|  | rs6976053 | | 7 | | ACHE | | T | | RR interval | | NA | | 1.96E-08 | |
|  |  | |  | |  | |  | | Pulse rate | | -0.02826 | | 1.67E-29 | |
|  | rs6486122 | | 11 | | ARNTL | | T | | Body mass index | | -0.01455 | | 2.12E-08 | |
|  |  | |  | |  | |  | | Worry too long after embarrassment | | 0.008065 | | 1.30E-09 | |
|  |  | |  | |  | |  | | Age at menarche | | 0.033 | | 9.30E-10 | |

Abbreviation: SNP, single nucleotide polymorphism; Chr, chromosome; EA, effect allele.
